# Cell death and antioxidant responses in *Mytilus galloprovincialis* under heat stress: Evidence of genetic loci potentially associated with thermal resilience

**DOI:** 10.1371/journal.pone.0321682

**Published:** 2025-04-23

**Authors:** Dimitrios K. Papadopoulos, Basile Michaelidis, Ioannis A. Giantsis

**Affiliations:** 1 Department of Zoology, Faculty of Sciences, School of Biology, Laboratory of Animal Physiology, Aristotle University of Thessaloniki, Thessaloniki, Greece; 2 Faculty of Agriculture, Forestry and Natural Environment, Laboratory of Ichthyology & Fisheries, Aristotle University of Thessaloniki, Thessaloniki, Greece; Bigelow Laboratory for Ocean Sciences, UNITED STATES OF AMERICA

## Abstract

The global seawater temperature is expected to further rise in the following years. While species have historically adapted to climatic variations, the current pace of climate change may exceed their ability to adapt. The abnormally increased seawater temperatures occasionally lead to high mortalities of marine bivalve mollusks, threatening the productivity of aquaculture and the sustainability of wild populations. This study investigates the antioxidant and cell death mechanisms of the Mediterranean mussel *Mytilus galloprovincialis* during a 25-day exposure to temperatures of 24°C, 26°C, and 28°C, by analyzing the transcription of key genes and assessing the oxidative damage on days 1, 3, 12, and 25. In addition, individuals resilient (survived at 28°C until day 30) and susceptible (died early at 26°C and 28°C) to thermal stress were collected to investigate potential polymorphisms in associated genes. The results showed increased transcription of antioxidant genes at higher temperatures. Elevated pro-apoptotic indices were initially observed at 26°C and a higher mortality than at 28°C. However, final mortality was much higher at 28°C. At 26°C, mussels exhibited the highest oxidative damage and pro-apoptotic indices after 25 days. At 28°C, although oxidative damage occurred after 24 hours, survivors maintained a prolonged activated antioxidant defense and increased *lc3b* transcription, which likely contributed to the observed reduction of pro-apoptotic and oxidative damage metrics on day 25, compared to 26°C. Further, the coding sequences of *catalase*, intracellular *Cu-Zn superoxide dismutase* (*Cu-Zn sod*), and *fas-associated protein with death domain* (*fadd*) from heat-resilient and heat-susceptible mussels were analyzed. Based on statistical correlation of nucleotide and genotype frequencies with resilience phenotypes, two novel single nucleotide polymorphisms (SNPs) in *Cu-Zn sod* and one in *fadd* were detected, potentially correlating with thermal stress resilience. These findings offer valuable insights into the physiological and genetic adaptations of *M. galloprovincialis* to rising temperatures and highlight loci potentially linking to thermal resilience.

## Introduction

Global mean sea surface temperature has increased from the beginning of the 20th century by about 0.88°C [1]. According to the recent report of the Intergovernmental Panel on Climate Change (IPCC), by 2100 the global mean sea surface temperature could increase by 1.4–4.4°C relative to the years 1850–1900, while by 2040 the temperature increase will almost certainly exceed 1.5°C [2]. As a result of global warming, extreme events such as marine heat waves have become more frequent and intense, posing significant threats to aquatic organisms [3]. These periods of significantly elevated seawater temperatures lasting from days to months, often cause severe problems to aquatic ecosystems and their services [4]. Increased seawater temperatures trigger significant bivalve mortalities in aquaculture farms and in the natural fisheries [5] and thus represent a limiting factor for their productivity.

Aquatic organisms are resilient to temperature fluctuations up to a certain tolerance range [6]. Temperature extremes in Northern Greece, with sea surface temperatures up to 29.5–30°C during 3 consecutive summers (2020–2022), caused almost 100% mortality in mussel farms [7], a situation which is repeated every year, with records from 2024 indicating a mass mortality in the end of August attributed to seawater temperatures higher than 31°C. Accordingly, the Pacific oyster *Crassostrea gigas*, another major aquaculture product, faces repeated summer mortalities worldwide for over five decades [8]. The different species have historically coped with climate shifts over their evolutionary timeline. However, the rapid pace of contemporary climate change could challenge their ability to adapt to such rapid changes [9] threatening their sustainability besides their productivity. In an effort to address this issue, selective breeding programs for thermal tolerance are being conducted globally [10,11] in bivalve aquaculture.

Among the stressors that aquatic ectotherms face, temperature is believed to be the major factor that drives selection pressure due to its profound impact on biochemistry and physiology [12]. Prolonged exposure to sublethal temperatures requires specific and reversible readjustments and thus physiological and behavioral tradeoffs of individuals to tolerate such conditions. Selection pressure under harsh conditions can lead to adaptations that have a genetic background. For instance, *M. galloprovincialis* exhibits increased heat tolerance and higher invasion potential compared to multiple congeneric species [13]. Although there are several studies on the genetic structure of bivalve populations from marine areas impacted by warming, e.g., [14], only a few inferences have been associated with genetic adaptation to temperature extremes. More specifically, Delorme et al. [15] demonstrated that thermal resilience in *Perna canaliculus* is influenced by genetic differences, with survival correlating with the differential regulation of the *hsp70* gene following heat stress, while a synonymous single noucleotide polymorphism (SNP) was correlated with the upper thermal limit of *Mytilisepta virgata* [16]. Other studies have identified SNPs linking to thermal resilience in antioxidant genes. Wang et al. [17] validated SNPs in the *Cu-Zn sod* coding region of *C. gigas* populations, while Li et al. [12] detected a non-synonymous SNP in *Mn-SOD* with differing genotype frequencies and transcriptional responses to heat stress in two oyster subspecies. Moreover, a non-synonymous SNP in SOD was linked to thermal adaptation in *Pinctada margaritifera* populations from contrasting thermal regimes [18]. In environments where changes are too rapid and intense, adaptive genetic variation might be favored [19]. The effort to appraise the engagement of temporal acclimation and genetics to thermal resilience is difficult but also highly important for applying efficient conservation techniques.

During normal conditions, reactive oxygen species (ROS) are removed effectively by the antioxidant defense system of the organism that maintains a balance between formation and elimination of ROS. The antioxidant machinery includes antioxidant enzymes such as superoxide dismutases and catalase, along with low molecular weight scavengers such as glutathione and metallothioneins. Higher levels of ROS are usually generated within the cells under stressful conditions when the antioxidant defense fails to maintain the redox balance. Evidence shows that reactive oxygen species may increase in marine bivalves after heat stress [20–22] and may also decrease presumably owing to a decrease of several biochemical pathways due to thermal stress [23]. Although ROS are implicated in multiple physiological processes [24] they can harm the macromolecules such as lipids, DNA, and proteins and cause cellular dysfunctions at high levels [25]. Peroxidation of lipids is a common form of cellular damage caused by oxidative stress. The reaction of ROS with lipids and lipoproteins alters the physical and chemical properties of the cell membranes [26] affecting vital cellular functions [27]. Thus, the dynamics of antioxidant mechanisms and the oxidative damage deserve special attention when studying the effects of elevated temperatures.

Superoxide dismutases (SODs; EC 1.15.1.1) represent a crucial defense against ROS [28] since they accelerate the reaction that converts the superoxide anion (O_2_^•−^) to O_2_ and hydrogen peroxide H_2_O_2_ [27]. Hydrogen peroxide is also harmful to the cell but it is decomposed by other enzymes such as catalase (EC 1.11.1.6). Superoxide dismutases are crucial to the survival of bivalves under heat stress [29]. Three main types of SOD are met in multi-cellular organisms. These are the intracellular cytosolic Cu/Zn-SOD (ic Cu/Zn-SOD), the extracellular Cu/Zn-SOD (EC-SOD), and the mitochondrial Mn-SOD [18]. Great attention has been paid to icCu/Zn-SOD due to its multiple functions, except for superoxide dismutation [30].

Excessive ROS within cells may result in oxidative stress and apoptotic signal induction [31]. Recently, an one week exposure of *Crassostrea virginica* to increasing temperatures was found to induce ROS-mediated oxidative damage leading to apoptosis [32,33]. Apoptosis is a highly conserved process of programmed cell death that occurs in multi-cellular organisms to maintain their homeostasis. Two major distinct pathways are implicated in apoptosis, in particular the death receptor-mediated (extrinsic) pathway, and the mitochondrial (intrinsic) pathway. The B cell lymphoma 2 (BCL-2) protein family is a diverse group of about 25 globular proteins [34] which are key regulators of the mitochondrial apoptotic pathway. Concerning their functions, BCL-2 members may be categorized as either pro-apoptotic or anti-apoptotic. Both categories are important for maintaining tissue homeostasis [35]. The pro-apoptotic regulator BAX competes with the anti-apoptotic BCL2 for the activation of caspases, a family of proteases which are important for the initiation of apoptosis. The FADD (Fas-Associated protein with Death Domain) protein is a key adaptor protein of the death receptor-mediated (extrinsic) pathway of apoptosis playing a crucial role in the signaling cascade that activates caspases to execute the apoptotic processes. FADD protein contains binding sites for procaspase-8 and procaspase-10 [36,37]. Recent studies have documented the increase of apoptotic indices in various bivalves after both acute [38,39] and longer thermal stress [32,33,39,40].

Autophagy is a cellular procedure that degrades and recycles damaged or unnecessary cellular components, including proteins and organelles, through lysosomal digestion. This mechanism primarily promotes survival under nutrient deficiency and stress. Excessive autophagy may lead to cell death and interact with apoptotic pathways [41]. LC3B (Microtubule-associated protein 1A/1B-light chain 3B) is an essential protein in autophagy as it assists in the formation of autophagosomes. Autophagosomes are important for the sequestration and degradation of misfolded proteins and damaged organelles. In invertebrates, such as the sea urchin (*Paracentrotus lividus*), LC3B-mediated autophagy is crucial for stress response, to sustain cell homeostasis helping the organism to survive in unfavorable conditions by supplying nutrients and energy through the recycling of cellular components [42]. Heat stress and oxidative damage have been shown to initiate the autophagic process in *M. galloprovincialis* [43], as well as in other bivalve species [44].

This study focused on the investigation of the physiological response of the Mediterranean mussel *Mytilus galloprovincialis* after exposure to persistently increased temperatures, up to its upper tolerance limit. Heat stress leads to the accumulation of reactive oxygen species (ROS) in bivalves, which, through their interplay with apoptotic pathways, is hypothesized to result in the differential transcription of genes associated with antioxidant defense and cell death responses in *M. galloprovincialis*, as well as in increased oxidative damage. To test this hypothesis, the expression of key genes (*bax*, *bcl-2*, *fadd*, *lc3b*, *Cu-Zn sod*, *catalase*) at 24°C, 26°C, and 28°C was studied in comparison to the control temperature of 18°C, and the oxidative damage was evaluated through the quantification of lipid peroxidation during a 25-day period.

A second hypothesis posits that recent temperature extremes in Northern Greece, coupled with high mussel mortalities, may have driven the prevalence of specific mutations in key genes involved in thermal stress response pathways. Therefore, the coding sequences of *catalase*, *intracellular Cu-Zn sod*, and *fadd* from heat-resilient (survived at 28°C until day 30) and heat-susceptible mussels (died early at 26°C and 28°C) were amplified to identify polymorphisms potentially associated with heat resilience. Further, 3D structures of the corresponding peptides were obtained to identify the region affected by the polymorphism and evaluate the potential influence on the peptide function. To our knowledge, this is the first attempt to investigate the transcriptional regulation of *lc3b* and *fadd* in bivalves exposed to increased temperatures and to identify thermal resilience-associated loci directly in the aforementioned genes of *M. galloprovincialis*. This study investigates the acute, intermediate and prolonged physiological responses of *M. galloprovincialis* to increasing temperatures and identifies genetic loci potentially contributing to thermal resilience, providing valuable insights into physiological and genetic adaptations under global warming.

## Materials and methods

### Animal acclimation and routine operations

In total, 700 *Mytilus galloprovincialis* mussels (wet weight 17.44 ± 2.11 g) were obtained evenly from two mussel farms in Northern Greece (Vistonikos Bay and Thermaikos Gulf) in late April 2021. No further permission was required, since mussels originated from farms and no endangered animal species was included in our study. Low genetic differentiation has been observed recently in *M. galloprovincialis* populations in the Aegean Sea [45] and thus the two mussel populations used are expected to be genetically homogenous. Shells were thoroughly cleaned under tap water and mussels were randomly divided into two aerated tanks containing 2.000 liters of filtered (sand filter, 20μm) natural seawater of 34.3‰ salinity and 17.8°C temperature. Tanks were equipped with a recirculating water system along with mechanical and biological filters, and a UV lamp. After two days, 670 animals were randomly distributed among eight 1000-litre tanks containing also natural seawater (equipped with the same recirculating system) in duplicate as follows: 50, 70, 90, and 125 mussels. Biofilters were fully capable of complete nitrification.

Mussels were acclimated in the tank conditions for two weeks before any other treatment. Temperature (Hanna HI98129, Hanna Instruments Inc., Woonsocket, RI, USA), and dissolved oxygen (Hanna HI9142), were measured once daily using electronic devices, while pH, total ammonia nitrogen (TAN), nitrites (NO_2_^-^) and nitrates (NO_3_^-^) were also recorded daily through saltwater test kits (Tetra, Melle, Germany). Mussels were fed on the live microalgae *Tisochrysis lutea* (CCAP 927/14) and *Tetraselmis* spp. (Mediteranean strain) at 1:1 dry weight ratio. The weekly amount of (dry) microalgae weight was 3% of the live weight of the mussels. Artificial light was provided by fluorescent lamps and a photoperiod of 16:8 h light:dark was set. Water temperature was 17.7 ± 0.68°C and all the nitrogen compounds were kept at safe levels. Each week, 5–15% of the water was exchanged for fresh seawater, depending on the mussel density in each tank. This practice ensured fixed water quality across all treatments and low nitrogen compound concentrations (TAN and NO_2_^-^ < 0.25 mg/L, NO_3_^-^ < 25 mg/L) throughout the experiment.

### Trial setup and samplings

After the acclimation phase, temperature was adjusted by electronic aquarium heaters (Digital Aquarium Heater DR-9300, Boyu, Guangdong Province, China) at a rate of 0.1°C per hour to reach 24°C, 26°C, and 28°C. More specifically, the two tanks containing 50 mussels were designated as the control tanks (18°C). In the remaining six tanks, temperature was adjusted to 24°C (70 mussel tanks), 26°C (90 mussel tanks), or 28°C (125 mussel tanks). The control temperature was selected to match the average seawater temperature in Northern Greece on May [46]. The average sea surface temperature in August in North Aegean is typically around 26°C and the mean maximum recorded temperatures on this month are about 28°C [46].

To ensure adequate numbers of mussels on the latter stages of the experiment, higher densities were used at higher temperatures, while lower densities were maintained at 18°C as minimal mortality was expected. Water quality was controlled with mature biofilters, daily waste removal, and consistent monitoring of pH, salinity, nitrogen compounds, and dissolved oxygen. All water quality parameters did not significantly varied across the different tanks except for dissolved oxygen concentration which had a maximum 1.09-fold difference among 18°C and 28°C treatments. The minimum mean dissolved oxygen concentration at 28°C was measured at 6.5 mg/L, never dropping below 6 mg/L, indicating supersaturation conditions, given that the maximum oxygen solubility at 28°C and 33–35‰ salinity is approximately 6 mg/L (YSI Oxygen Solubility Table). Considering the low stocking density and the optimized water quality management, it was hypothesized that the density had no significant impact on the mussels’ temperature responses.

After the intended temperature was attained in each tank (day 0), four samplings were performed on day 1 (24 hours), day 3, day 12, and day 25. During the experiment mussels were fed as in the acclimation phase and water parameters were measured once a day. Three mussels were randomly collected from each tank at all sampling points, thus six animals from each treatment. The mantle tissue was dissected with sterilized tools, placed in 1.5 ml tubes and was immediately flash frozen on liquid nitrogen. All samples were then stored at -80°C. Tanks were checked continuously for dead animals. Mussels were considered dead when their valves were open and remained open after multiple mechanical stimuli.

### Samplings of animals for genotyping

**Fig 1:**
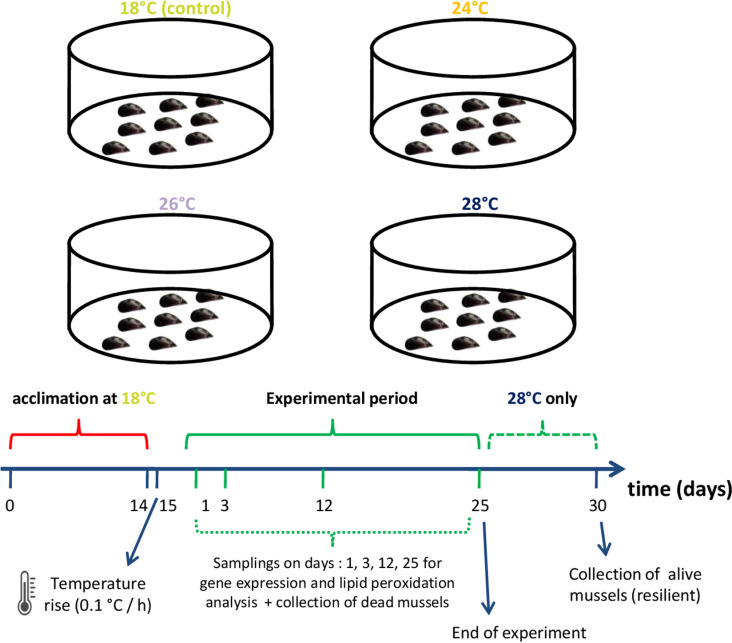
Summary of the experimental design.

Resilient and susceptible to thermal stress mussels were collected for sequencing of key genes that are implicated in apoptosis and antioxidant defense. The aim was to investigate polymorphisms within the coding regions that may be associated with heat stress resilience. Twenty alive mussels were randomly collected from the two treatments of 28°C on day 30, when their mortality reached 81%. These mussels were designated as the resilient ones. Dead mussels were gathered during the experimental trial, and twenty of them were marked as the susceptible animals. More specifically, sixteen mussels from the 28°C treatments that died within days 5–8 (before the exponential phase of the mortality curve at 28°C) corresponding to two dead mussels from each replicate per day, and four mussels from 26°C on days 5 and 6 when the mortality at 26°C equaled and exceeded the mortality at 28°C. Mantle tissue of all the above animals was dissected with sterilized tools, flash frozen in liquid nitrogen, and stored at -80°C. A summary of the experimental design is shown in Fig 1.

### RNA extraction and cDNA synthesis

RNA extractions were performed from the animals sampled for gene expression analysis and from the resilient and susceptible animals as well. Totally, 136 samples of *M. galloprovincialis* mantle tissue (96 for gene expression and 40 for genotyping) were subjected to RNA extraction using the NucleoZOL reagent (Macherey-Nagel, Düren, Germany) according to the protocol of the manufacturer. RNA purity and concentration were assessed on a Quawell UV-Vis 5000 spectrophotometer (Quawell Technology, San Jose, CA, USA), and the extracted RNA was stored at −80°C until the reverse transcription. RNA samples with an A_260_/A_280_ ratio outside the range of 1.8–2, indicating low quality, were discarded. In the reverse transcription step, cDNA was produced using the PrimeScript RT Reagent Kit (Takara, Otsu, Japan) and the oligodT primers, following the protocol provided by the manufacturer. cDNA was diluted and samples were stored at −20°C until the application of PCRs.

### Quantification of gene expression

The gene expression profile of six genes (Table 1) was evaluated through real-time quantitative PCR (qPCR). PCR primers were either obtained from other studies or were designed from the selected sequences using the *primer3* software (Version 4.1.0.) [47]. Primer dimmers and hairpin formation were predicted in the software *Oligo Analyzer 1.0.2.* When efficiency was lower than 90% or higher than 110% the primer pair was replaced. Melt curves were also examined after each assay to ensure the absence of double peaks and primer dimmers. Threshold cycle (C_t_) values of the target genes were normalized to the C_t_ values of the reference gene (β-actin) as the different temperatures were not altering its expression. The relative expression of the target genes was calculated through the comparative C_t_ (2^–ΔΔCt^) method as described in Livak and Schmittgen [48] since efficiencies of all primer pairs were not more than 5% different from the reference gene’s. The primers for *lc3b* were designed within conserved regions after multiple sequence alignments from different bivalve species [e.g., *Crassostrea gigas* (XM_011417532.3) and *Mytilus trossulus* (XM_063570666.1)] (Table 1) as no sequence for *M. galloprovincialis* was available in public databases.

**Table 1:** Primer sequences, PCR efficiencies (*E*), annealing temperature, amplicon size, and target sequence accession number of the genes analyzed in the qPCR.

Gene	Forward primer (5’-3’) Reverse primer (5’-3’)	*E* (%)	Annealing	Amplicon (bp)	GenBank accession number	Reference
B-cell lymphoma 2 (*bcl2)*	TGCCACCATCACAAATTCAGC GCCACAATCCTTCCCCAGTT	95.2%	55°C	187	KC545829.1	This study
Bcl-2-associated X protein (*bax*)	CCAACAGGTCCACCATTAGAAC CTCTTGGCCACAGTTAGGAATG	97.9%	53°C	152	KC545830.1	[50]
Microtubule associated protein 1 light chain 3 beta (*lc3b*)	CCWCAAGARCTCTCYATGTC TCYTGTGAKGCATAWGTCAT	103.2%	53°C	188	XM_011417532.3 XM_063570666.1	This study
Fas-associated protein with death domain (*fadd*)	CAGTCAAGAGAAAAACTGACAGC GGCTGAGCTTGTTGTAAAGCC	98.1%	57°C	203	KF051276.1	This study
Cu/Zn superoxide dismutase (*Cu/Zn-sod*)	GCCAATGCAGAGGGAAAAGC ATGACACCACAAGCCAGACGA	96.8%	56°C	182	FM177867.1	This study
Catalase (*catalase*)	CTCTGACCGTGGAACCCCTGA ATCACGGATGGCATAATCTGGA	101.6%	55°C	193	AY743716.2	[51]
*β-actin*	CGACTCTGGAGATGGTGTCA GCGGTGGTTGTGAATGAGTA	98.9%	58°C	153	AF157491.1	[52]

The quantification of the expression in the different temperatures was calculated in relation to the expression of the same gene in the control treatment (18°C). For each gene, 80 samples (5 mussels/treatment/sampling time) were run in duplicate using the KAPA SYBR® FAST qPCR Master Mix (2×) kit in 10 μL final volume. PCR reactions were carried out in a qPCR Thermocycler Eco 48 Real-time PCR (Illumina, San Diego, CA, USA). Each reaction well contained 10 ng cDNA, 5 μL of KAPA SYBR® FAST qPCR Master Mix, 2 μΜ of each primer, and PCR-grade water up to 10 μL. Details concerning the primers are summarized in Table 1. Initial denaturation step was 3 min at 95°C and extension step was 72°C for 15 sec. Annealing temperatures are shown in Table 1. Amplification efficiency for each primer pair was calculated in preliminary trials (S1 Table), using 5 random samples, by five-fold dilutions of 5 cDNA samples as E = 10 ^(−1/slope)^ [49]. The slope was generated from the regression line of five serial five-fold dilutions and the corresponding C_t_ values versus the relative concentration of cDNA.

### Sequencing of target genes

Since mussels of the genus *Mytilus* are known for cross-hybridization, the mitochondrial COI fragment was sequenced in all sampled mussels for verification of their identity using the universal primers designed by Folmer et al. [53]. Approximately 4% of the sampled mussels were excluded from the analysis and replaced due to significant genetic differences from the rest of the individuals. Amplifications of the coding sequences (cds) of *catalase*, intracellular *Cu/Zn superoxide dismutase* (*Cu/Zn sod*), and *fas-associated protein with death domain* (*fadd*) genes, were made through conventional PCR in the Thermocycler FastGene ULTRA Cycler Gradient (NIPPON Genetics EUROPE, Düren, Germany). PCR reactions were conducted in 20 μL volume and each PCR tube contained 10 μL of FastGene Taq 2 × Ready Mix (NIPPON Genetics EUROPE, Düren, Germany), 0.4 μΜ of primers, 30 ng of cDNA from heat-resilient and heat-susceptible individuals, and PCR-grade water up to 20 μL. The thermal cycling program was an initial denaturation step of 95°C for 3 min, followed by 38 cycles of 95°C for 30 sec, primer annealing at 52–55°C (Table 2) for 30 sec, and an extension step at 72°C for 30–90 seconds depending on the size of the target. A final extension stage of 10 min at 72°C was also implemented in all assays.

**Table 2:** Primers and annealing temperature for the amplification of genes/gene fragments.

Gene	Forward primer (5’-3’) Reverse primer (5’-3’)	Annealing	Amplicon size (bp)	GenBank accession number
*Catalase*	ACAGCCACAATGACAGTTGGAC GTCCGTATTCTGGATCAGCCT	55°C	1357	KX957929.1
*Cu/Zn-sod*	CTCTATAGGTATGGCAGCTAA GAAATTCCAATGACACCACAA	52°C	474	AJ581746.1
*Fadd*	GGGAGACATGGAATTTAACTCG TCCCTTTTCAAGTTTTGATGC	53°C	703	KF051276.1

Five (5) μL of the PCR products were used for electrophoresis on 1.2% agarose gel stained with Midori Green Advance (NIPPON Genetics EUROPE, Düren, Germany) and then agarose gels were observed under UV light. Properly amplified products were purified using the NucleoSpin Gel and PCR Clean-up® kit (Macherey Nagel, Düren, Germany). The purified products were then sequenced in both directions in an ABI 3730xl automatic sequencer. The obtained chromatograms were visually checked and then analyzed using Finch TV 1.4.0 (Geospiza, Seattle, WA, USA) and BioEdit [54] software. All sequences were aligned in MEGA X [55] through the MUSCLE algorithm.

### Protein annotation and structure

The purpose of this analysis was to identify the region affected by the detected polymorphisms and evaluate their putative impact on peptide function. The obtained sequences were translated into amino acids in MEGA X and the corresponding amino acid sequences were uploaded to I-TASSER online server [56]. The three-dimensional (3D) protein structures were generated by I-TASSER and the best-supported structure, identified by the highest C-score [57] was visualized and further processed through the molecular graphic software PyMOL version 3.0.1. Protein domain annotation as well as ligand binding sites, active sites, etc. identification was based on I-TASSER predictions and was also validated from annotations of homologous proteins of related bivalve species in GenBank.

### TBARS assay

TBARS (Thiobarbituric Acid Reactive Substances) assay measures lipid peroxidation by detecting malondialdehyde (MDA), a product of oxidative degradiation of lipids. The purpose of this assay is to assess the level of oxidative damage in biological samples. Fifty milligrams (50 mg) of mantle tissue were manually homogenized in 500 μl of ice-cold phosphate-buffered saline (PBS, pH 7.4) containing 0.137 M NaCl, 2.7 mM KCl, 10 mM Na_2_HPO_4_, 1.8 mM KH_2_PO_4_ using a hand tissue grinder. Both Bradford and lipid peroxidation assays were estimated from these tissue homogenates. All samples were placed inside a box full of ice during the homogenization. Samples were mixed by vortex for 10 sec and then centrifuged at 3000 x g for 10 min at 4°C. The supernatant was transferred to new tubes and stored at -80°C until further analyses. Bradford assay [58] was performed to evaluate the protein concentration of each homogenate.

ROS are hard to measure because they are highly reactive with extremely short lifespan. Their cellular damage was estimated through the assessing the lipid peroxidation which was calculated by measuring the quantity of malondialdehyde (MDA), as it is a specific end-product of the oxidative degradation of lipids and is more stable than ROS. MDA standard curve was made by measuring the absorbance of several dilutions (0–2.4 nmol) of malondialdehyde tetrabutylammonium salt (Sigma, St. Louis, MO, USA) dissolved in absolute ethanol (PanReac AppliChem, Darmstadt, Germany). MDA was used along with ultrapure water and a mixture of 20% trichloroacetic acid (TCA, PanReac AppliChem, Darmstadt, Germany) and 0.5% thiobarbituric acid (TBA, Sigma, St. Louis, MO, USA). 3 mM butylated hydroxytoluene (BHT, Glentham Life Sciences, Corsham, UK) was also added. Samples were incubated in a dry bath (100°C) for 30 minutes, cooled at room temperature, and then centrifuged at 3000 x g for 10 min. 900 μl of each sample were transferred to a cuvette and absorbance was measured at 532 nm in a spectrophotometer (M501 single beam, CAMSPEC, Leeds, United Kingdom). MDA concentration in the samples was measured accordingly. BHT was included in the assay to prevent further peroxidation of the homogenate during heating. Each sample contained 600 μl TCA 20%-TBA 0.5%, 20μl of 150 mM BHT (3 Mm), 20–30 μl of the sample (corresponding to 50 μg of proteins) and ultrapure water up to 1 ml. MDA content and corresponding TBARS levels were calculated as described in Sachett et al. [59]. The amount of MDA was calculated using a molar extinction coefficient of 1.56 x 10^5^ M^-1^ cm^-1^ and TBARS levels were expressed as nmoles of TBARS per mg of proteins in the sample.

### Statistical analysis

Due to the limited capacity of the RT-PCR plate (48-well format), quantitative PCRs could not be performed simultaneously. Therefore, gene expression analysis was conducted within a given sampling time. For consistency, TBARS levels were also compared within a given sampling time. Prior to statistical analysis, all data were tested for normality using the Shapiro-Wilk test. Data transformation of *catalase* transcription followed to meet the assumptions of normality using a square root transformation and subsequently, all data were analyzed using the single factor one-way analysis of variance (ANOVA) at *p* < 0.05 (S6 Table). By running ANOVA, the test of Brown-Forsythe (at p < 0.05) was also checked to confirm the homogeneity of variances. When statistically significant differences were found among the different temperature treatments, Tukey’s HSD multiple post-hoc comparisons were conducted to define which groups differ (*p* < 0.05). Statistical analyses and figures were made in GraphPad Prism (Version 10.4.1). All the results were expressed as means ± standard deviation.

PCA analysis was conducted in GraphPad Prism (Version 10.4.1). The method for selecting principal components was based on the percentage of total explained variance which was set at 75%. The corresponding PC scores plot and factor loading plot were also made and visualized in GraphPad Prism. The PC scores PC1, PC2, and PC3 were subsequently subjected to PERMANOVA analysis in R software (Version 4.4.2) using the Euclideian distances and 999 permutations. Finally, statistical analysis of allele and genotype frequencies in resilient and susceptible mussels was performed using the chi-squared (χ^2^) test. Results were considered statistically significant when *p* values were lower than 0.05.

## Results

Since environmental variables (such as oxygen levels, pH, and feeding) that could influence the outcomes were continuously monitored (S2 Table), it is inferred that the observed results were predominantly driven by temperature. As mentioned earlier, low genetic differentiation was recently observed in *M. galloprovincialis* populations in the Aegean Sea [45]. However, to evaluate the potential influence of mussel origin on the results, the animals were divided so that each tank contained an equal number of mussels from Vistonikos Bay and Thermaikos Gulf, placed in separate mesh baskets. Mortality generally did not differ by origin. Gene expression and TBARS levels also exhibited small variation, as all analyzed indices had little standard deviations, and no evident origin-dependent patterns (S3, S4 Tables). The analyzed indices consistently had values close to the mean with limited dispersion. Furthermore, the selected resistant and susceptible individuals were evenly obtained from all associated tanks and mesh baskets.

### Mortality

**Fig 2:**
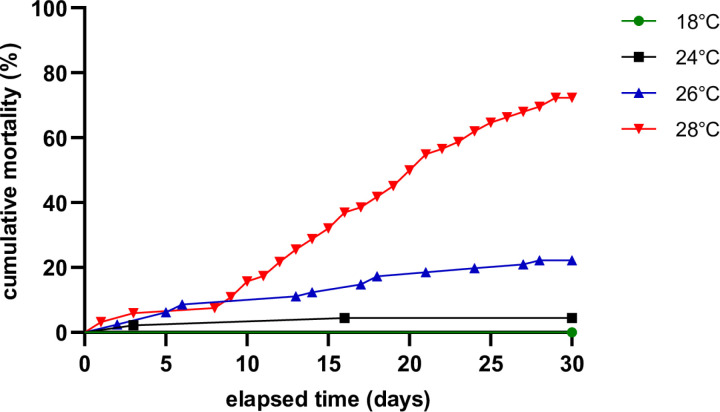
Mortality in the different temperatures throughout the experimental period.

Mussel mortality was zero in the control group. At 24°C, the final mortality was 4.44%, and no dead mussel was recorded after day 9 (Fig 2). At 26 °C, the death percentage reached 20.72%, and half of the dead animals passed away within the first 6 days. Total mortality at 28°C was 72.2% on day 25 and reached its exponential phase on day 8 (Fig 2).

### Transcription of genes involved in apoptotic processes

**Fig 3:**
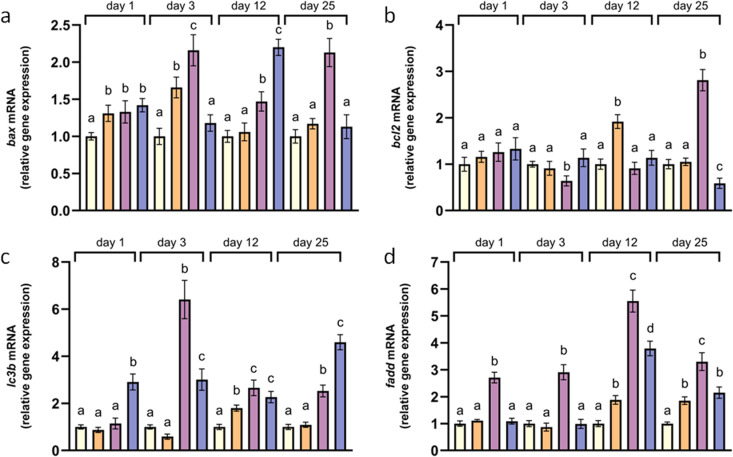
Relative mRNA levels of bax (a), *bcl2* (b), *lc3b* (c), and *fadd* (d) in the mantles of *Mytilus galloprovincialis.* Relative expression was calculated relative to the control (18°C) within each sampling time. Values are means ± SD, **n** = 5 different mussels. Different lowercase letters represent statistically significant differences in means (*p* < 0.05) within a given sampling time. Light yellow represents the control treatment, orange corresponds to 24°C, purple to 26°C, and blue to 28°C.

Considering the gene transcription analyses, of the six sampled mussels in each group, one was excluded from the analyses based on RNA quality and the A_260_/A_280_ ratio. Specifically, in 3 out of 16 groups (3 samples out of 96), one RNA was discarded due to low quality. In the remaining 13 groups, one sample was randomly excluded to have equal sample sizes across all groups. The expression of *bax* in mussel mantles at 24°C was found significantly higher from18°C on days 1 and 3, and later returned to control levels (Fig 3a). In addition, the mRNA levels of *bcl2* and *lc3b* was generally similar to controls throughout the exposure at 24°C, and increased only on day 12 (Figs 3b and 3c). The transcription of *fadd* at 24°C was at first equal to the control mussels and was found significantly increased later on days 12 and 25 (Fig 3d). In mussels reared at 26°C, the transcription of *bax*, *lc3b*, and *fadd* was significantly higher from the control at all sampling times compared to the control temperature (Figs 3a and 3c).The mRNA levels of *bcl2* were equal or lower from the mussels at 18°C until day 12, and exhibited a significant elevation on day 25. Concerning the mussels exposed to 28°C, *bax* transcription was initially slightly increased in comparison with the controls, peaking on day 12, while equal to 18°C transcription was found on day 25 (Fig 3a). The mRNA of *bcl2* remained unchanged relative to the controls until day 25, when significantly reduced levels were detected (Fig 3b). At 28°C, the transcription of *lc3b* and *fadd* was significantly elevated throughout the experiment (Figs 3b and 3c).

### Transcription of antioxidant genes

**Fig 4:**
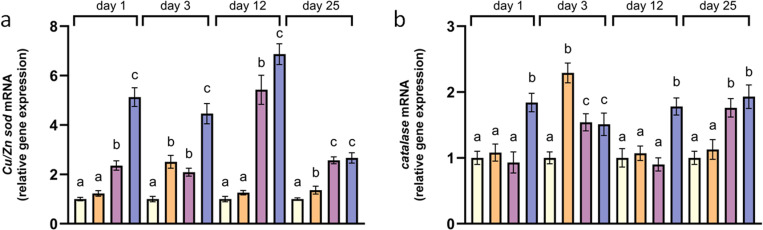
Relative mRNA levels of *Cu/Zn sod* (a), and catalase (b) in the mantles of *Mytilus galloprovincialis.* Relative expression was calculated relative to the control (18°C) within each sampling time. Values are means ± SD, **n** = 5 different mussels. Different lowercase letters represent statistically significant differences in means (*p* < 0.05) within a given sampling time. Light yellow represents the control treatment, orange corresponds to 24°C, purple to 26°C, and blue to 28°C.

At 24°C, both *Cu-Zn sod* and *catalase* mRNA levels were significantly elevated compared to the control mussels 3 days after the temperature increase. Concerning the rest of the sampling times, the transcription of both antioxidant genes in the mantles of mussels at 24°C did not differed from controls, except for day 25 when *Cu-Zn sod* transcription was significantly higher (Figs 4a and 4b). At 26°C, the transcription of *Cu-Zn sod* was constantly higher compared to the control, while *catalase* transcription was increased or equal to mussels at 18°C (Figs 4a and 4b). Finally, a persistent significantly increased transcription of both antioxidant genes was observed in mussels reared at 28°C compared to the control mussels (Figs 4a and 4b).

### Lipid peroxidation

**Fig 5:**
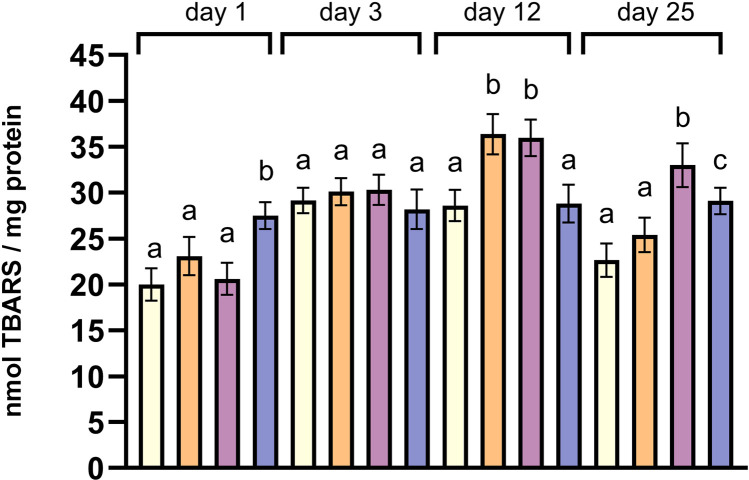
TBARS concentration in the mantles of mussels in the different temperatures. Values are means ± SD, **n** = 5 different mussels. Different lowercase letters represent statistically significant differences in means (*p* < 0.05) within a given sampling time. Light yellow represents the control treatment, orange corresponds to 24°C, purple to 26°C, and blue to 28°C.

On day 1, a significantly increased level of lipid peroxidation was found only in the mantles of mussels exposed to 28°C as witnessed by the increased TBARS concentration (Fig 5). On day 3, all treatments exhibited comparable TBARS concentrations. Later, on day 12, significantly higher peroxidation was detected at 24°C and 26°C in comparison with the controls and 28°C, which had equal levels of TBARS. In the last sampling time, on day 25, control mussels and those at 24°C had similar TBARS levels while significantly increased levels were found at 26°C and 28°C, and the highest levels were detected in the mantles of mussels at 26°C (Fig 5).

### Multivariate analysis

**Fig 6:**
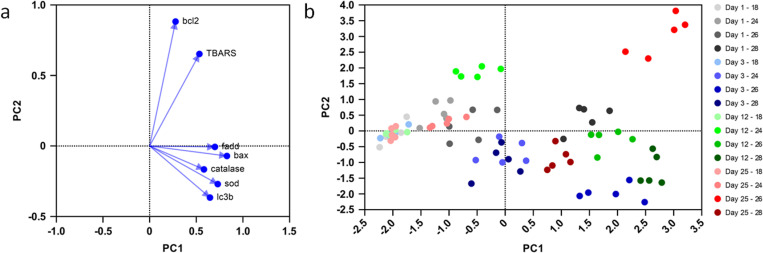
Factor loading plot for PC1 and PC2, illustrating the contribution of variables to the principal components **(a)**. Scatterplot of PC scores for the first two principal components (PC1 and PC2) derived from PCA **(b)**. PC1 explains 40.4% while PC2 20.61% of the variance. The PCA was generated from the complete dataset of gene transcription and TBARS measurements.

PCA results showed that the first three principal components (PCs) explain 75.92% of the data variance. More specifically PC1 (40.4%), PC2 (20.61%) and PC3 (14.91%) were selected for analysis. The corresponding eigenvalues were 2.83, 1.44, and 1.04 respectively. All factors showed a positive correlation with PC1 scores (Fig 6a). TBARS and *bcl2* were strongly associated with PC2, showing high positive loadings, while *catalase*, *Cu-Zn sod*, *bax*, *fadd*, and *lc3b* were clustered near PC1, indicating a, more or less, shared contribution to this component. Interestingly, both the apoptotic genes (*bax*, *fadd*) and the antioxidant genes (*catalase*, *sod*) along with *lc3b* displayed strong positive correlations and thus are closely related in their contribution to the principal components (Fig 6a).

The scatterplot of PC scores for PC1 and PC2 shows that all control samples (18°C) are tightly clustered (Fig 6b), which was expected since their mean was always set to 1, and all calculations were made relative to them. Regarding the mussels at 24°C, those sampled on days 1, 12 and 25 were placed closely, whereas mussels from day 3, were clustered with the 28°C samples taken on the same day. Mussels exposed to 26°C exhibited significant variability across sampling times and thus were placed at completely distinct positions, while those at 28°C also showed high variability between sampling times, with day 12 exhibiting great differences (Fig 6b).

According to the perMANOVA results (Table 3), temperature had the strongest effect on the PC scores, explaining 47.9% of the total variance (R² = 0.479), showing in parallel highly significant results (*p* < 0.001). Time alone explains 14.6% of the variance (R² = 0.146) and is also highly significant (*p* < 0.001). The interaction between time and temperature explains one third of the variance (R² = 0.33) and is also highly significant (*p* < 0.001), indicating that the effect of temperature varies across time. The residual variance was only 4% of the total (R² = 0.04), suggesting that the model used, captures most of the variation in the data.

**Table 3:** PERMANOVA results using the principal components PC1, PC2, PC3: Effects of time, temperature, and their interaction on Euclidean distances.

	Df	Sums Of Sqs	Mean Sqs	F. Model	R^2^	*p*-value
time	3	61.44	20.48	71.68	0.146	0.001***
temperature	3	201.3	67.1	234.85	0.479	0.001***
time: temperature	9	138.81	15.42	53.98	0.33	0.001***
residuals	64	18.29	0.28		0.04	
Total	79	419.84			1	

The method used was Euclideian distances with 999 permutations.

### Polymorphisms and distribution in resilient and susceptible mussels, and peptide 3D structure

Several substitutions were detected in the amplified coding sequences of *fadd*, *Cu/Zn-sod*, and *catalase*. All non-synonymous SNPs, and the synonymous SNPs that had significantly differential distribution among resilient and susceptible mussels were included in the results.

#### FADD.

Two non-synonymous nucleotide substitutions were detected in the cds of *fadd*. The first (cds-544, GenBank Acc. No. KF051276) was a dimorphism caused by a G to A transition that results in a substitution of Val with Ile at position 182 of the signal peptide. The allele G was significantly associated with increased susceptibility to thermal stress (*p* < 0.05, Table 4). The second polymorphism (cds-550) was also a dimorphism caused by an A to T transversion leading to the substitution of Thr from Ser at position 184 in the signal peptide. Allele and genotype frequencies did not differ and thus no association was made with thermal stress resilience (Table 4).

**Table 4:** Number of polymorphic alleles and corresponding genotypes in the coding sequences (cds) of *fadd*, *Cu/Zn-sod*, and *catalase* in resilient and susceptible *Mytilus galloprovincialis.*

Position	Genotypes	Susceptible	Resilient	Mutation type	Allele	Susceptible	Resilient
*fadd*							
**cds-544** ^b^	G/G	12	6	Val (V) to Ile (I) GTA or ATA	G A	30 10	21 19
	G/A	6	9				
	A/A	2	5				
**cds-550**	A/A	8	9	Thr (T) to Ser (S) ACA or TCA	A T	20 18	23 15
	A/T	4	5				
	T/T	7	5				
*Cu-Zn sod*							
cds-195^b^	T/T	18	7	Synonymous substitution TCA or TCT	T A	36 2	15 21
	A/T	0	1				
	A/A	1	10				
cds-285 ^b^	T/T	12	8	Asp (D) to Glu (E) GAT or GAG	T G	31 7	20 16
	T/G	7	4				
	G/G	0	6				
*catalase*							
**cds-1198**	C/C	11	13	Pro (P) to Ser (S) CCA or TCA	C T	27 7	27 7
	C/T	5	1				
	T/T	1	3				

The values of χ^2^ and *p* from the chi-squared tests and the percentages (%) of each genotype and allele in resilient and susceptible groups are shown in S5 Table.

^a^Bolded positions affect important amino acids, based on protein annotation.

^b^Indicates significant differences between resilient and susceptible animals. Τhe specific differences are underlined.

**Fig 7:**
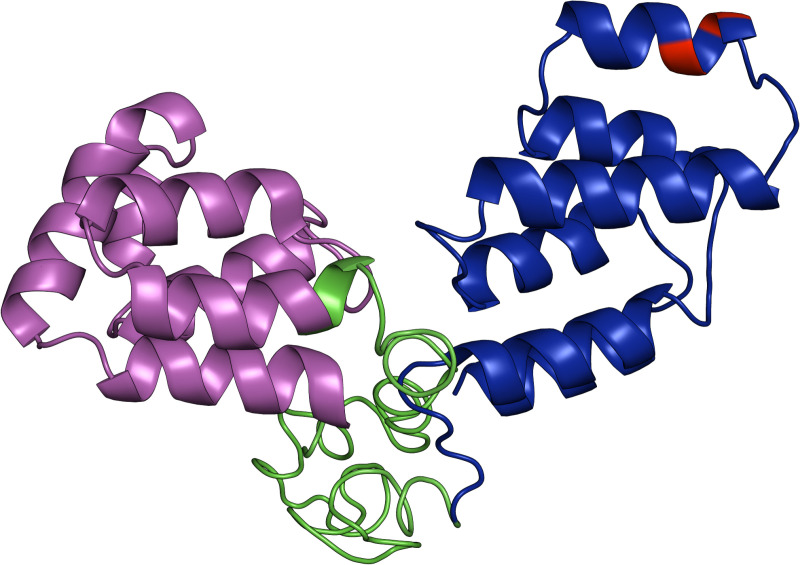
The three-dimensional (3D) structure of FADD (C-score **=-1.99).** The death effector domain (DED) is shown in magenta. Τhe death domain (DD) of protein- protein interactions appears in blue and the rest of the peptide is shown in green. The amino acids affected by the polymorphisms are shown in red.

The 3D structure of *Mytilus galloprovincialis* Fas-associated protein with death domain (FADD) is shown in Fig 7. Based on the annotation of the same protein in GenBank (AHI17305.1) the death effector domain (DED) is colored in magenta and the death domain (DD) of protein-protein interactions appears in blue. The two identified amino acid substitutions are both located in the latter (red color) which directly contributes to the protein function.

#### Cu-Zn SOD.

One non-synonymous substitution was detected in the cds of the intracellular *Cu-Zn sod*. In addition, one synonymous substitution with significant differences in the allele and genotype frequencies between resilient and susceptible mussels was also identified (Table 4). The former SNP (cds-285, GenBank Acc. No. FM177867) was generated due to a dimorphism originating from a T to G transversion which results in the substitution of Asp to Glu at position 95 in the corresponding peptide. Both allele and genotype frequencies exhibited highly significant differences (S5 Table) among resilient and susceptible individuals. Thus the G allele, the G/G genotype and the Glu residue at this position, were associated with increased susceptibility to increased temperatures. The synonymous substitution at cds-195 also exhibited highly significant differences and the prevalence of T and the T/T genotype were correlated with increased susceptibility to chronic thermal stress.

**Fig 8:**
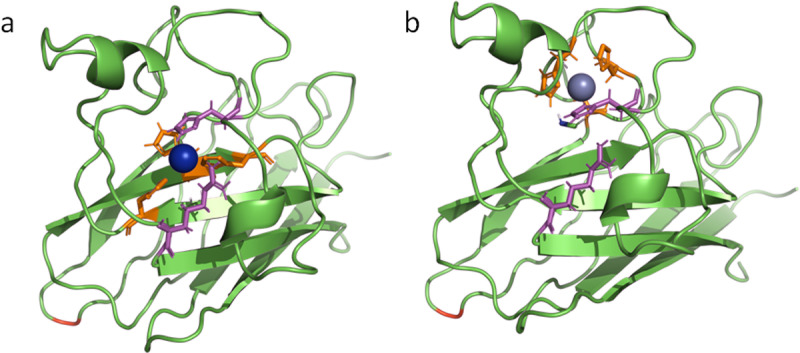
The three-dimensional (3D) structure of Cu/Zn SOD (C-score **=** **1.35).** The Cu-binding site (**a**, C-score=0.96) and the Zn-binding site (**b**, C-score=0.53) are shown in the different captures in orange and the catalytic region is indicated in magenta. The amino acid that is affected by the substitution appears in red.

The 3D structure of the *Mytilus galloprovincialis* Cu-Zn SOD monomer is shown in Fig 8. The amino acids that are responsible for Cu and Zn binding (orange) as well as the active site residues (magenta) have been depicted as sticks (Figs 8a and 8b). As shown in both Figures the amino acid that is affected (red) by the substitution is possibly not central to the main catalytic function of the enzyme.

#### Catalase.

Concerning the amplified sequence of *catalase*, only one non-synonymous substitution was identified. It was caused by a transition of C to T at position 1198 of our amplified region (Table 4). This transition leads to a substitution of Pro with Ser at position 449 of the signal peptide in *Mytilus coruscus* whose sequence is full in GenBank (KX957929.1). No difference was found in allele and genotype frequencies of susceptible and resilient mussels.

**Fig 9:**
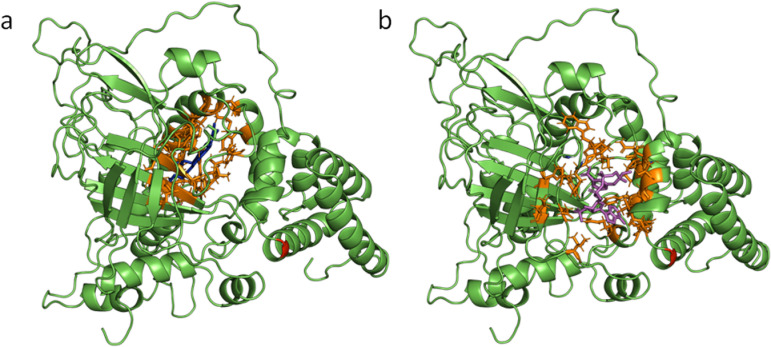
The three-dimensional (3D) structure of the catalase monomer (C-score **=** **1.11).** Heme binding site (**a**, C-score=0.93) is shown in blue and NADPH binding site (**b,** C-score=0.96) is displayed in magenta and the corresponding important amino acids are shown as sticks in orange. The amino acid that is affected by the polymorphism appears in red.

The 3D structure of the monomer *Mytilus galloprovincialis* Catalase is shown in Fig 9. The amino acids that are responsible for heme and NADPH binding have been illustrated as sticks (Figs 9a and 9b, respectively). As appears in both Figures the amino acid that change (red) due to the substitution is not located close to any important region of the enzyme.

## Discussion

This study examined the antioxidant defense mechanisms and cell death responses in *Mytilus galloprovincialis* exposed to persistently elevated temperatures. By assessing key gene transcription patterns and lipid peroxidation, we aimed to elucidate the molecular responses associated with thermal stress. Additionally, we investigated potential genetic polymorphisms that may associate with adaptive genetic variation under recent temperature extremes in Northern Greece. Our findings offer valuable insights into the mechanisms of thermal resilience, highlighting adaptive strategies and identifying polymorphic loci that could contribute in future aquaculture and/or conservation practices in the face of rapidly changing environmental conditions. Our findings support the hypothesis that increasing temperatures lead to differential transcription of genes related to antioxidant defense and cell death pathways in *M. galloprovincialis*, as well as to oxidative damage.

Multivariate analysis identified temperature as the most influential factor, explaining 47.9% of the variability in the indices. The PCA plot revealed distinct clustering of the 26°C and 28°C treatments, particularly on days 12 and 25, emphasizing the stronger impact of elevated temperatures over time. Additionally, time alone accounted for 14.6% of the variance, while the interaction between time and temperature explained 33%. These findings suggest that both prolonged exposure and increasing temperature significantly influence the measured indices, with effects becoming more evident at higher temperatures.

Severe mortalities were observed at 28°C reaching 81% after 30 days, which is in general in agreement with previous studies involving mussels from the same area, exposed to similar temperatures [60]. Mortality until day 15 was approximately 30%, while discrepancies in the final mortality at 28°C compared to Anestis et al. [60], whose experimentation involved similar temperatures and mussels from the same area, may be attributed to higher temperature fluctuations, which sometimes were measured at 28.4°C or 28.5°C. In the 24°C and 26°C treatments, the reported mortalities by Anestis et al. [60] were almost identical to this study.

### Antioxidant defense and lipid peroxidation

Increasing temperatures have been linked with the up-regulation of genes implicated in the antioxidant defense in bivalves [61–63]. Temperature stress has been reported to lead to up-regulated transcription of *sod* and *catalase* genes and to increased activities of the corresponding enzymes in *Crassostrea gigas, Mytilus galloprovincialis* and *Katelysia rhytiphora* [61] after 14 days of exposure, evidencing an activated antioxidant defense to counteract the increased ROS production. Nevertheless, if ROS proliferation from thermal stress is not eliminated, oxidative damage will appear [64]. In this study, *M. galloprovincialis* exhibited similar to the control or occasionally increased transcription of both antioxidant genes at 24°C, while at 26°C, the transcription of *Cu-Zn sod* was consistently elevated throughout the experiment. Exposure to 28°C, also enhanced the transcription of both antioxidant genes throughout the experiment, while mRNA levels were always higher or similar to the levels of mussels at 26°C suggesting an increasing antioxidant defense in accordance to the temperature rise.

When the antioxidant machinery cannot detoxify the excessive ROS generated by thermal stress, lipid peroxidation occurs [65]. TBARS levels at 24°C exceeded the levels of the control treatment only on day 12, while at 26°C oxidative damage was observed after prolonged (days 12 and 25) exposure. Interestingly, at 28°C oxidative damage was observed only on day 1 and on day 25, while on day 25, lipid peroxidation levels were lower from the levels of mussels at 26°C. The sustained highly upregulated antioxidant response as was evidenced by elevated transcription of *Cu-Zn sod* and *catalase* in the surviving mussels at 28°C (27.8% survival on day 25), may have contributed to the reduced oxidative damage compared to 26°C after 25 days of exposure. In accordance to these findings, one and two-week exposure of *Scrobicularia plana* to 25°C led to increased SOD activity that protected the organism from cellular damage since it was accompanied by similar to control (17°C) or slightly declined MDA levels [66]. Furthermore, unchanged or reduced peroxidation of lipids was observed after 4–6 days of a heatwave simulation at temperatures of 22°C and 25°C in the clams *Ruditapes decussatus* and *Ruditapes philippinarum* [67,68].

### Apoptosis and autophagy

Apoptosis and autophagy can be activated by ROS [31] as well as by up-regulated genes linked with the respiratory chain and especially with ROS formation [69]. Based on the factor loadings plot, our results also show that antioxidant defense, apoptotic, and autophagic pathways probably respond together under thermal stress. In addition, the proximity of *Cu-Zn sod* and *catalase* with *fadd* and *bax* could support the interplay between apoptosis and antioxidant defenses. Velez et al. [70] reported a 17-fold increased expression of *bax* in the gills of *R. phillipinarum* after 28 days of exposure to 25°C when compared to specimens at 17°C and 21°C. Georgoulis et al. [63] also found enhancement of *bax* mRNA in *Ostrea edulis* in accordance to the magnitude of the thermal stress. In this work, mussels at 24°C exhibited minor fluctuations of *bax* and *bcl2*, while those at 26°C, showed a constantly elevated *bax* mRNA, with a peak on days 3 and 25, when the expression exceeded the expression of mussels at 28°C. Accordingly, *bcl2* transcription decreased only on day 3 at 26°C. This initial up-regulation of pro-apoptotic indices and the decrease of *bcl2* mRNA which could reflect the downregulation of cellular survival mechanisms and may be associated with the higher mortality at 26°C compared to 28°C until day 6. Mussels at 26°C appeared to experience significant oxidative stress after chronic exposure, as indicated by the highest levels of *bax* and *fadd* mRNA among the treatments, as well as by the highest TBARS levels on day 25. This suggests that prolonged exposure to 26°C, disrupted the balance between oxidative damage and cellular defense mechanisms, resulting in heightened pro-apoptotic activity and oxidative damage compared to 28°C. The temperature of 26°C may represent a transitional temperature where stress is significant enough to overwhelm the defenses after chronic exposure but not extreme enough to trigger the more robust adaptive mechanisms seen at 28°C. The few surviving mussels at 28°C (27.8%) may have coped more efficiently with chronic thermal stress, likely due to a better physiological response that was likely supported by a more robust genetic background.

Autophagy can delay apoptosis by providing cells with nutrients and energy through recycling [71, 72]. Looking at the PCA’s factor loadings plot, the proximity of *lc3b* to the antioxidant genes and the apoptotic genes suggests *lc3b* induction as a stress response mechanism. In *Mytilus edulis* the transcriptional up-regulation of pro-apoptotic genes during hypoxia was coupled with an induction of autophagic enzyme activity, while *Crassostrea gigas* which is more tolerant to hypoxia, exhibited a simultaneously muted apoptotic and autophagic response [44]. At 24°C *lc3b* transcription was found generally equal to 18°C. However, exposure of *M. galloprovincialis* at 26°C or 28°C resulted in increased transcription of *lc3b* throughout the exposure. Thus, mussels seem to employ autophagy to sustain their energy reserves against demanding environments as other bivalves do when exposed to hostile conditions such as starvation [73] and hypersaline environments [74]. The higher levels of TBARS and apoptosis-promoting genes of mussels at 26°C, on day 25, compared to mussels at 28°C were accompanied by lower *lc3b* transcription at 26°C, indicating that surviving mussels under long-term exposure at 28°C might have also relied on autophagy to delay apoptosis and increase survival.

The pro-apoptotic protein BAX and the anti-apoptotic protein BCL2 are involved in the intrinsic mitochondrial pathway of apoptosis and compete to regulate the activation of caspases. FADD is primarily implicated in the extrinsic apoptotic pathway. The over-expression of a *fadd* gene in *R.phillipinarum* was found to promote the apoptosis of cells [37]. In this study, the exposure of mussels at 24°C led to a late and slight transcriptional up-regulation of *fadd*. Interestingly, at 26°C mussels exhibited increased mRNA levels of f*add* throughout the experimental period that were always higher than the levels of mussels at 28°C. Mussels at 28°C, exhibited upregulated transcription of *fadd* only on days 12 and 25 compared to the control mussels.

The executioner caspase-3 transcription and apoptosis were induced after thermal stress in previous studies on mussels of the genus *Mytilus* [75,76]. Recently, apoptosis-related and antioxidant defense genes were both found significantly upregulated in *Scapharca broughtonii* after acute and chronic thermal stress [39]. In the present study, the antioxidant defense was activated at the highest temperature from the beginning of the trial. The magnitude of the heat stress in the case of 26°C seems to have elevated the cellular damage from day 12 and led to the activation of pathways associated with cell death. In parallel, the potentially elevated autophagy (increased *lc3b* transcription) compared to 18°C and 24°C may play part in delaying apoptosis and increasing survival at this temperature. However, the excessive autophagy that was observed even after medium heat stress, could lead to tissue degradation, reduced growth, and poor meat quality [77], impacting farming practices in warming environments.

The examined markers could help differentiate the acute and chronic stress responses. Exposure at 26°C was identified as the most stressful in the acute phase, as it resulted in higher mortality than at 28°C, and was accompanied by upregulation of pro-apotpotic (*bax*, *fadd*) and downregulation of anti-apoptotic (*bcl2)* markers. Furthermore, 26°C appears to be nearly as stressful as 28°C during chronic temperature due to prolonged oxidative damage and sustained pro-apoptotic activity. Finally, 28°C proved highly stressful in the chronic phase, resulting in significant mortality, which was accompanied by downregulation of *bcl2* and upregulation of *lc3b* in long-term survivors.

### Genetic polymorphisms and protein structures

FADD protein comprises an N-terminal death effector domain (DED) and a C-terminal death domain (DD). The DD is connected to the receptor, while the DED contains the binding site for procaspase-8 and procaspase-10 [36,37]. Two non-synonymous substitutions were detected in the *fadd* gene of *M. galloprovincialis*. One was differentially distributed between resistant and susceptible mussels and was found to affect an amino acid within the DD. Another non-synonymous substitution with differential genotype and allele distribution was identified in *Cu/Zn sod*. This substitution was found to affect an amino acid not directly involved in the primary functional regions of the corresponding peptide. However, that kind of amino acid substitutions can still have profound effects on the structure, stability, interactions, and function of the protein [78,79]. The single non-synonymous substitution identified in *catalase* exhibited similar allele and genotype frequency between resilient and susceptible mussels.

Amino acid substitutions within important stress-responsive proteins such as the superoxide dismutases maybe the result of adaptation to the variable marine environment. For instance, Wang et al. [17] validated the presence of SNPs in the coding region of *Cu-Zn sod* after examining three different populations of the Pacific oyster *C. gigas* from China. In addition, Li et al. [12] exposed two oyster subspecies from distinct ecological niches to acute heat shock and detected a non-synonymous SNP with dissimilar genotype frequencies in Mn-SOD which exhibited differentially induced transcription following the heat stress. A non-synonymous substitution with differing frequency and expression was found in SOD genes of two *Pinctada margaritifera* populations inhabiting environments of contrasting thermal regimes [18]. In *Argopecten irradians*, a non-synonymous substitution in the cds of the extracellular Cu-Zn SOD was associated with the resistance of this scallop to *Vibrio anguillarum* infections [80].

A single synonymous substitution was detected in our work with significant differences of allele and genotype frequencies in the intracellular Cu-Zn SOD. Although synonymous substitutions were traditionally believed to be “silent” and have no effect on phenotype, at present we know that they can have significant impacts through various processes at the levels of transcription, translation, and mRNA stability [81,82]. Synonymous mutations in specific genetic regions have been evidenced to impact fitness and play a role in adaptive responses [83]. Moreover, a synonymous substitution was recently correlated with the physiological upper thermal limit of the mussel *Mytilisepta virgata* [16].

## Conclusion

Investigating responses to stressful environments under selection pressure is of great importance for understanding the underlying molecular and phenotypic mechanisms involved in the tolerance or the susceptibility of an organism. Recent extreme temperature events in the Mediterranean, particularly in Northern Greece caused mortalities that reached 100% in mussel aquaculture farms [7] highlighting the vulnerability of *M. galloprovincialis* to rapid environmental changes. Such events may promote the prevalence of specific polymorphisms, like those identified in this study. Moreover, frequent exposure and survival under abiotic stressors can lead to locally adapted genotypes as observed in mussel populations [84].

In the context of global warming, it is important to explore how natural populations adapt to changing environments. Besides the physiological responses of *Mytilus galloprovincialis* to increased temperatures based on mRNA levels and lipid peroxidation assays, this study identified genetic loci potentially involved in thermal stress resilience*.* Future research should explore the physiological response incorporating protein-level analyses and further investigate the association of identified polymorphisms with heat resilience. Expanding the dataset with more individual sequences would also strengthen the conclusions regarding the identified SNPs. The interactive effects of several factors such as the proliferation of pathogens and harmful algal blooms, promoted by ocean warming, create a complex interplay with varying impacts on bivalves. Thus, further experiments, especially multi-factorial ones and field surveys are needed to better understand the global effects of rising temperatures on bivalves in the context of climate change.

## Supporting information

S1 Table Amplification efficiency of primer pairs, calculated from preliminary trials using five-fold dilutions of five random samples. (DOCX)

S2 Table Water parameters during the experimental period. Values are mean ± SD.
(DOCX)

S3 Table Mean values and standard deviations of all measurements and Shapiro-Wilk test results for normality assessment. (DOCX)

S4 Table Fold change values from gene transcription analysis and TBARS levels of all mussels. Mann-Whitney U tests (data not shown) comparing the two areas of origin (Thermaikos Gulf = T and Vistonikos Bay = V) at each sampling time and temperature indicate no statistically significant differences in any of the indices, suggesting no effect of area of origin.
(DOCX)

S5 Table The percentages of the different alleles and genotypes in resilient and susceptible mussels and χ^2^ and *p* values from the chi-squared tests on their distribution among resilient and susceptible individuals. Statistically significant differences (*p* < 0.05) are indicated with bold and an asterisk (*).
(DOCX)

S6 Table Results of one-way ANOVA and Tukey’s post-hoc comparisons, along with Brown-Forsythe test results for homogeneity of variances. For the Brown-Forsythe’s test the test statistic, degrees of freedom (numerator, denominator), and *p*-value are shown. For ANOVA the test statistic, *p*-value, and R^2^ are shown along with the *p*-values from Tukey’s multiple post-hoc comparisons among treatments.
(DOCX)
