# Supplementary material for: Cell death and antioxidant responses in Mytilus galloprovincialis under heat stress: Evidence of genetic loci potentially associated with thermal resilience
Source: PLoS One. 2025 Apr 23;20(4):e0321682. doi: 10.1371/journal.pone.0321682 (PMC12017574; doi:10.1371/journal.pone.0321682)
Supplement: S1 Table — (DOCX) [file pone.0321682.s001.docx]

|  | B-cell lymphoma 2 **(*bcl2)*** | | | | |
| --- | --- | --- | --- | --- | --- |
| Sample | 1 | 2 | 3 | 4 | 5 |
| **slope** | -3.414 | -3.315 | -3.592 | -3.446 | -3.462 |
| ***E*** | 1.963 | 2.003 | 1.898 | 1.951 | 1.945 |
| **Mean *E%*** | **95.2** | | | | |
|  | Bcl-2-associated X protein **(*bax*)** | | | | |
| Sample | 1 | 2 | 3 | 4 | 5 |
| **slope** | -3.562 | -3.385 | -3.42 | -3.224 | -3,296 |
| ***E*** | 1.909 | 1.974 | 1.961 | 2.043 | 2.011 |
| **Mean *E%*** | **97.9** | | | | |
|  | Microtubule associated protein 1 light chain 3 beta **(*lc3b*)** | | | | |
| Sample | 1 | 2 | 3 | 4 | 5 |
| **slope** | -3.38 | -3.145 | -3.303 | -3.187 | -3.241 |
| ***E*** | 1.976 | 2.08 | 2.008 | 2.06 | 2.035 |
| **Mean *E%*** | **103.2** | | | | |
|  | fas-associated protein with death domain **(*fadd*)** | | | | |
| Sample | 1 | 2 | 3 | 4 | 5 |
| **slope** | -3.254 | -3.299 | -3.471 | -3.392 | -3.445 |
| ***E*** | 2.029 | 2.01 | 1.941 | 1.972 | 1.951 |
| **Mean *E%*** | **98.1** | | | | |
|  | Cu/Zn superoxide dismutase **(*Cu/Zn-SOD*)** | | | | |
| Sample | 1 | 2 | 3 | 4 | 5 |
| **slope** | -3.523 | -3.451 | -3.279 | -3.356 | -3.411 |
| ***E*** | 1.922 | 1.949 | 2.018 | 1.986 | 1.964 |
| **Mean *E%*** | **96.8** | | | | |
|  | Catalase **(*catalase*)** | | | | |
| Sample | 1 | 2 | 3 | 4 | 5 |
| **slope** | -3.302 | -3.182 | -3.24 | -3.376 | -3.331 |
| ***E*** | 2.008 | 2.062 | 2.035 | 1.978 | 1.996 |
| **Mean *E%*** | **101.6** | | | | |
|  | *β-actin* | | | | |
| Sample | 1 | 2 | 3 | 4 | 5 |
| **slope** | -3.276 | -3.226 | -3.444 | -3.491 | -3.335 |
| ***E*** | 2.02 | 2.042 | 1.951 | 1.934 | 1.995 |
| **Mean *E*%** | **98.9** | | | | |

**S1 Table.** **Amplification efficiency of primer pairs, calculated from preliminary trials using five-fold dilutions of five random samples.**

^a^Initial cDNA quantity in all samples was 200 ng and the 5 five-fold dilutions were 200, 40, 8, 1.6, and 0.32 ng. Log quantities of cDNA (2.3, 1.6, 0.9, 0.2, and -0.49 respectively) were plotted against Ct values and slopes were calculated from the regression line for each sample.

^b^*E* = 10^(-1/slope)^.

^c^*E*% = (*E-1*) x 100% , not shown in Table. Mean *E*% = mean E% value of the five samples.
