# Supplementary material for: Cell death and antioxidant responses in Mytilus galloprovincialis under heat stress: Evidence of genetic loci potentially associated with thermal resilience
Source: PLoS One. 2025 Apr 23;20(4):e0321682. doi: 10.1371/journal.pone.0321682 (PMC12017574; doi:10.1371/journal.pone.0321682)
Supplement: S2 Table — Values are mean ± SD. (DOCX) [file pone.0321682.s002.docx]

| Tank | Temperature (ᵒC) | Dissolved Oxygen (mg/L) | pH |
| --- | --- | --- | --- |
| 1 | 17.9±0.41 | 7.1±0.32 | 8.26±0.17 |
| 2 | 17.7±0.54 | 7±0.59 | 8.29±0.19 |
| 3 | 24.2±0.21 | 6.9±0.44 | 8.25±0.12 |
| 4 | 24.3±0.25 | 6.8±0.61 | 8.21±0.16 |
| 5 | 26.2±0.31 | 6.8±0.34 | 8.18±0.09 |
| 6 | 25.9±0.38 | 6.8±0.45 | 8.18±0.1 |
| 7 | 28.2±0.33 | 6.5±0.32 | 8.15±0.13 |
| 8 | 27.9±0.4 | 6.6±0.23 | 8.14±0.15 |

**S2 Table.** **Water quality parameters in the different treatments during the experimental period.** Values are mean ± SD.
