# Supplementary material for: Cell death and antioxidant responses in Mytilus galloprovincialis under heat stress: Evidence of genetic loci potentially associated with thermal resilience
Source: PLoS One. 2025 Apr 23;20(4):e0321682. doi: 10.1371/journal.pone.0321682 (PMC12017574; doi:10.1371/journal.pone.0321682)
Supplement: S3 Table — (DOCX) [file pone.0321682.s003.docx]

| **treatment**  **S3 Table.** **Mean values and standard deviations of all measurements and Shapiro-Wilk test results for normality assessment.** | **bax** | | **Shapiro-Wilk test** | | **bcl2** | | **Shapiro-Wilk test** | | |
| --- | --- | --- | --- | --- | --- | --- | --- | --- | --- |
|  | mean | SD | W statistic | p value | mean | SD | W statistic | p value | |
| Day 1 18°C | 1.00 | 0.05 | 0.89 | 0.4 | 1.00 | 0.15 | 0.86 | 0.23 | |
| Day 1 24°C | 1.31 | 0.11 | 0.98 | 0.94 | 1.16 | 0.12 | 0.93 | 0.6 | |
| Day 1 26°C | 1.33 | 0.15 | 0.99 | 0.98 | 1.26 | 0.2 | 0.98 | 0.93 | |
| Day 1 28°C | 1.42 | 0.09 | 0.83 | 0.15 | 1.33 | 0.24 | 0.91 | 0.48 | |
| Day 3 18°C | 1.00 | 0.11 | 0.83 | 0.15 | 1.00 | 0.06 | 0.88 | 0.32 | |
| Day 3 24°C | 1.66 | 0.14 | 0.94 | 0.68 | 0.91 | 0.15 | 0.89 | 0.40 | |
| Day 3 26°C | 2.16 | 0.21 | 0.95 | 0.78 | 0.64 | 0.11 | 0.83 | 0.14 | |
| Day 3 28°C | 1.18 | 0.11 | 0.97 | 0.88 | 1.14 | 0.19 | 0.99 | 0.99 | |
| Day 12 18°C | 1.00 | 0.08 | 0.92 | 0.57 | 1.00 | 0.11 | 0.83 | 0.15 | |
| Day 12 24°C | 1.06 | 0.12 | 0.92 | 0.53 | 1.92 | 0.15 | 0.85 | 0.21 | |
| Day 12 26°C | 1.47 | 0.13 | 0.84 | 0.18 | 0.91 | 0.13 | 0.91 | 0.46 | |
| Day 12 28°C | 2.20 | 0.11 | 0.83 | 0.15 | 1.14 | 0.16 | 0.88 | 0.32 | |
| Day 25 18°C | 1.00 | 0.09 | 0.83 | 0.15 | 1.00 | 0.1 | 0.83 | 0.14 | |
| Day 25 24°C | 1.17 | 0.07 | 0.87 | 0.3 | 1.05 | 0.08 | 0.83 | 0.14 | |
| Day 25 26°C | 2.13 | 0.19 | 0.83 | 0.15 | 2.81 | 0.23 | 0.9 | 0.46 | |
| Day 25 28°C | 1.13 | 0.16 | 0.85 | 0.21 | 0.59 | 0.11 | 0.87 | 0.29 | |
|  | **lc3b** | | **Shapiro-Wilk test** | | **fadd** | | **Shapiro-Wilk test** | | |
|  | mean | SD | W statistic | p value | mean | SD | W statistic | p value | |
| Day 1 18°C | 1.00 | 0.09 | 0.83 | 0.15 | 1.00 | 0.10 | 0.93 | 0.65 | |
| Day 1 24°C | 0.87 | 0.11 | 0.83 | 0.15 | 1.11 | 0.04 | 0.87 | 0.27 | |
| Day 1 26°C | 1.15 | 0.23 | 0.84 | 0.16 | 2.71 | 0.20 | 0.92 | 0.55 | |
| Day 1 28°C | 2.91 | 0.34 | 0.86 | 0.25 | 1.09 | 0.11 | 0.98 | 0.95 | |
| Day 3 18°C | 1.00 | 0.09 | 0.91 | 0.48 | 1.00 | 0.11 | 0.96 | 0.81 | |
| Day 3 24°C | 0.59 | 0.11 | 0.97 | 0.87 | 0.87 | 0.15 | 0.92 | 0.53 | |
| Day 3 26°C | 6.41 | 0.81 | 0.85 | 0.22 | 2.91 | 0.28 | 0.97 | 0.89 | |
| Day 3 28°C | 3.01 | 0.45 | 0.84 | 0.18 | 0.99 | 0.17 | 0.89 | 0.37 | |
| Day 12 18°C | 1.00 | 0.11 | 0.96 | 0.83 | 1.00 | 0.11 | 0.93 | 0.6 | |
| Day 12 24°C | 1.80 | 0.13 | 0.84 | 0.18 | 1.88 | 0.16 | 0.93 | 0.58 | |
| Day 12 26°C | 2.66 | 0.33 | 0.98 | 0.97 | 5.55 | 0.41 | 0.79 | 0.06 | |
| Day 12 28°C | 2.27 | 0.24 | 0.86 | 0.24 | 3.79 | 0.27 | 0.86 | 0.23 | |
| Day 25 18°C | 1.00 | 0.11 | 0.94 | 0.68 | 1.00 | 0.06 | 0.93 | 0.58 | |
| Day 25 24°C | 1.09 | 0.11 | 0.83 | 0.15 | 1.85 | 0.14 | 0.98 | 0.94 | |
| Day 25 26°C | 2.53 | 0.25 | 0.86 | 0.23 | 3.30 | 0.33 | 0.94 | 0.66 | |
| Day 25 28°C | 4.60 | 0.32 | 0.89 | 0.37 | 2.15 | 0.21 | 0.93 | 0.61 | |
|  | **Cu-Zn sod** | | **Shapiro-Wilk test** | | **sqrt (catalase)** | | **Shapiro-Wilk test** | | |
|  | mean | SD | W statistic | p value | mean | SD | W statistic | | p value |
| Day 1 18°C | 1.00 | 0.07 | 0.92 | 0.53 | 1.00 | 0.10 | 0.93 | | 0.57 |
| Day 1 24°C | 1.23 | 0.11 | 0.91 | 0.45 | 1.08 | 0.13 | 0.88 | | 0.3 |
| Day 1 26°C | 2.36 | 0.19 | 0.88 | 0.31 | 0.93 | 0.16 | 0.93 | | 0.59 |
| Day 1 28°C | 5.13 | 0.38 | 0.87 | 0.26 | 1.84 | 0.14 | 0.79 | | 0.06 |
| Day 3 18°C | 1.00 | 0.11 | 0.91 | 0.48 | 1.00 | 0.09 | 0.97 | | 0.85 |
| Day 3 24°C | 2.51 | 0.26 | 0.91 | 0.47 | 2.29 | 0.15 | 0.98 | | 0.91 |
| Day 3 26°C | 2.09 | 0.16 | 0.85 | 0.21 | 1.54 | 0.13 | 0.92 | | 0.53 |
| Day 3 28°C | 4.46 | 0.41 | 0.9 | 0.43 | 1.51 | 0.17 | 0.92 | | 0.53 |
| Day 12 18°C | 1.00 | 0.11 | 0.98 | 0.95 | 1.00 | 0.14 | 0.86 | | 0.24 |
| Day 12 24°C | 1.26 | 0.09 | 0.9 | 0.42 | 1.07 | 0.11 | 0.92 | | 0.56 |
| Day 12 26°C | 5.43 | 0.59 | 0.81 | 0.11 | 0.90 | 0.10 | 0.85 | | 0.18 |
| Day 12 28°C | 6.87 | 0.42 | 0.89 | 0.36 | 1.78 | 0.13 | 0.98 | | 0.91 |
| Day 25 18°C | 1.00 | 0.05 | 0.87 | 0.25 | 1.00 | 0.10 | 0.96 | | 0.84 |
| Day 25 24°C | 1.36 | 0.16 | 0.93 | 0.58 | 1.13 | 0.15 | 0.93 | | 0.62 |
| Day 25 26°C | 2.57 | 0.14 | 0.95 | 0.72 | 1.76 | 0.14 | 0.95 | | 0.72 |
| Day 25 28°C | 2.67 | 0.21 | 0.95 | 0.73 | 1.93 | 0.18 | 0.96 | | 0.77 |
|  | **TBARS (fold change)** | | **Shapiro-Wilk test** | |  |  |  | |  |
|  | mean | SD | W statistic | p value |  |  |  | |  |
| Day 1 18°C | 1.00 | 0.09 | 0.79 | 0.06 |  |  |  | |  |
| Day 1 24°C | 1.15 | 0.05 | 0.96 | 0.81 |  |  |  | |  |
| Day 1 26°C | 1.03 | 0.09 | 0.91 | 0.49 |  |  |  | |  |
| Day 1 28°C | 1.38 | 0.07 | 0.94 | 0.7 |  |  |  | |  |
| Day 3 18°C | 1.00 | 0.05 | 0.96 | 0.8 |  |  |  | |  |
| Day 3 24°C | 1.03 | 0.05 | 0.97 | 0.88 |  |  |  | |  |
| Day 3 26°C | 1.04 | 0.06 | 0.96 | 0.77 |  |  |  | |  |
| Day 3 28°C | 0.97 | 0.07 | 0.94 | 0.63 |  |  |  | |  |
| Day 12 18°C | 1.00 | 0.06 | 0.97 | 0.89 |  |  |  | |  |
| Day 12 24°C | 1.27 | 0.08 | 0.98 | 0.93 |  |  |  | |  |
| Day 12 26°C | 1.26 | 0.07 | 0.98 | 0.94 |  |  |  | |  |
| Day 12 28°C | 1.01 | 0.07 | 0.91 | 0.45 |  |  |  | |  |
| Day 25 18°C | 1.00 | 0.08 | 0.82 | 0.12 |  |  |  | |  |
| Day 25 24°C | 1.12 | 0.07 | 0.94 | 0.67 |  |  |  | |  |
| Day 25 26°C | 1.46 | 0.10 | 0.94 | 0.67 |  |  |  | |  |
| Day 25 28°C | 1.28 | 0.06 | 0.95 | 0.77 |  |  |  | |  |
