# Supplementary material for: Cell death and antioxidant responses in Mytilus galloprovincialis under heat stress: Evidence of genetic loci potentially associated with thermal resilience
Source: PLoS One. 2025 Apr 23;20(4):e0321682. doi: 10.1371/journal.pone.0321682 (PMC12017574; doi:10.1371/journal.pone.0321682)
Supplement: S4 Table — Mann-Whitney U tests (data not shown) comparing the two areas of origin (Thermaikos Gulf = T and Vistonikos Bay = V) at each sampling time and temperature indicate no statistically significant differences in any of the indices, suggesting no effect of area of origin. (DOCX) [file pone.0321682.s004.docx]

**Table S4**. **Fold change values from gene transcription analysis, and TBARS levels of all mussels.** Mann-Whitney U tests (data not shown) comparing the two areas of origin (Thermaikos Gulf = T and Vistonikos Bay = V) at each sampling time and temperature indicate no statistically significant differences in any of the indices, suggesting no effect of area of origin.

| **Day** | **Temperature** | **Origin** | *bax* | *bcl2* | *lc3b* | *fadd* | *sod* | *catalase* | TBARS |
| --- | --- | --- | --- | --- | --- | --- | --- | --- | --- |
| 1 | 18°C | T | 0.96 | 0.91 | 0.92 | 1.15 | 1.11 | 1.08 | 20.95 |
| 1 | 18°C | T | 1.07 | 0.94 | 0.99 | 0.90 | 0.94 | 0.93 | 20.12 |
| 1 | 18°C | V | 0.98 | 0.87 | 0.99 | 1.04 | 1.02 | 0.87 | 20.55 |
| 1 | 18°C | V | 1.05 | 1.03 | 0.95 | 0.97 | 0.93 | 1.02 | 16.98 |
| 1 | 18°C | V | 0.94 | 1.25 | 1.15 | 0.94 | 1.00 | 1.10 | 21.40 |
| 1 | 24°C | T | 1.16 | 1.18 | 0.77 | 1.09 | 1.19 | 1.28 | 19.93 |
| 1 | 24°C | T | 1.33 | 0.99 | 0.86 | 1.18 | 1.06 | 1.03 | 25.46 |
| 1 | 24°C | T | 1.36 | 1.33 | 0.85 | 1.09 | 1.33 | 0.97 | 23.54 |
| 1 | 24°C | V | 1.23 | 1.15 | 0.81 | 1.12 | 1.25 | 0.98 | 24.10 |
| 1 | 24°C | V | 1.47 | 1.15 | 1.06 | 1.07 | 1.32 | 1.14 | 22.42 |
| 1 | 26°C | T | 1.25 | 1.53 | 0.94 | 2.43 | 2.17 | 0.91 | 21.35 |
| 1 | 26°C | T | 1.41 | 0.98 | 1.54 | 2.70 | 2.59 | 1.02 | 19.27 |
| 1 | 26°C | V | 1.30 | 1.21 | 1.03 | 2.99 | 2.22 | 0.79 | 19.06 |
| 1 | 26°C | V | 1.56 | 1.33 | 1.13 | 2.70 | 2.29 | 1.15 | 23.27 |
| 1 | 26°C | V | 1.12 | 1.25 | 1.11 | 2.74 | 2.53 | 0.77 | 20.15 |
| 1 | 28°C | T | 1.34 | 1.25 | 2.59 | 0.94 | 5.76 | 2.08 | 25.80 |
| 1 | 28°C | T | 1.37 | 1.55 | 2.82 | 1.13 | 5.02 | 1.84 | 27.16 |
| 1 | 28°C | T | 1.57 | 1.29 | 3.48 | 1.08 | 4.94 | 1.79 | 29.78 |
| 1 | 28°C | V | 1.41 | 1.57 | 2.73 | 1.06 | 5.15 | 1.74 | 27.76 |
| 1 | 28°C | V | 1.41 | 0.99 | 2.92 | 1.24 | 4.78 | 1.77 | 27.07 |
| 3 | 18°C | T | 0.90 | 0.94 | 1.14 | 0.92 | 1.11 | 0.94 | 30.16 |
| 3 | 18°C | T | 1.19 | 0.97 | 0.91 | 1.15 | 0.85 | 1.13 | 27.27 |
| 3 | 18°C | V | 0.94 | 1.10 | 1.04 | 0.87 | 1.04 | 1.02 | 30.61 |
| 3 | 18°C | V | 0.98 | 0.99 | 0.95 | 1.08 | 0.92 | 0.89 | 29.49 |
| 3 | 18°C | V | 0.99 | 1.00 | 0.96 | 0.98 | 1.08 | 1.01 | 28.23 |
| 3 | 24°C | T | 1.87 | 0.81 | 0.72 | 0.82 | 2.44 | 2.51 | 30.10 |
| 3 | 24°C | T | 1.72 | 1.03 | 0.56 | 0.84 | 2.65 | 2.35 | 31.87 |
| 3 | 24°C | T | 1.51 | 0.85 | 0.60 | 1.00 | 2.27 | 2.19 | 28.12 |
| 3 | 24°C | V | 1.58 | 0.76 | 0.64 | 1.02 | 2.89 | 2.29 | 29.30 |
| 3 | 24°C | V | 1.61 | 1.10 | 0.42 | 0.66 | 2.30 | 2.12 | 31.20 |
| 3 | 26°C | T | 2.46 | 0.57 | 7.77 | 3.18 | 1.92 | 1.37 | 30.75 |
| 3 | 26°C | T | 2.18 | 0.60 | 6.39 | 2.92 | 2.15 | 1.58 | 30.48 |
| 3 | 26°C | V | 2.19 | 0.77 | 5.66 | 2.83 | 2.27 | 1.68 | 32.70 |
| 3 | 26°C | V | 2.10 | 0.63 | 6.25 | 2.55 | 1.92 | 1.63 | 29.18 |
| 3 | 26°C | V | 1.88 | 0.63 | 5.99 | 3.06 | 2.19 | 1.43 | 28.39 |
| 3 | 28°C | T | 1.31 | 1.21 | 2.59 | 0.87 | 4.34 | 1.35 | 28.15 |
| 3 | 28°C | T | 1.21 | 1.40 | 2.77 | 1.21 | 4.09 | 1.40 | 29.43 |
| 3 | 28°C | T | 1.02 | 0.89 | 3.77 | 0.92 | 5.06 | 1.76 | 24.82 |
| 3 | 28°C | V | 1.23 | 1.06 | 2.77 | 1.13 | 4.13 | 1.45 | 30.57 |
| 3 | 28°C | V | 1.13 | 1.14 | 2.94 | 0.82 | 4.68 | 1.59 | 27.98 |
| 12 | 18°C | T | 0.92 | 0.90 | 1.01 | 1.18 | 1.15 | 0.97 | 30.63 |
| 12 | 18°C | T | 1.12 | 0.98 | 0.97 | 0.90 | 1.04 | 0.88 | 27.92 |
| 12 | 18°C | T | 0.93 | 0.99 | 0.85 | 1.00 | 0.93 | 1.01 | 28.27 |
| 12 | 18°C | V | 1.00 | 0.94 | 1.02 | 1.04 | 1.01 | 1.23 | 29.86 |
| 12 | 18°C | V | 1.03 | 1.19 | 1.15 | 0.88 | 0.87 | 0.91 | 26.31 |
| 12 | 24°C | T | 0.91 | 1.78 | 1.68 | 1.68 | 1.34 | 1.09 | 36.73 |
| 12 | 24°C | T | 1.15 | 1.91 | 1.73 | 2.00 | 1.33 | 0.92 | 37.76 |
| 12 | 24°C | V | 1.09 | 1.90 | 1.78 | 1.91 | 1.23 | 1.16 | 35.36 |
| 12 | 24°C | V | 0.96 | 2.17 | 1.79 | 1.75 | 1.28 | 0.99 | 33.18 |
| 12 | 24°C | V | 1.19 | 1.84 | 2.02 | 2.05 | 1.12 | 1.18 | 38.86 |
| 12 | 26°C | T | 1.35 | 0.93 | 2.63 | 5.83 | 5.07 | 0.84 | 33.25 |
| 12 | 26°C | T | 1.40 | 1.02 | 3.13 | 4.87 | 6.25 | 0.87 | 38.29 |
| 12 | 26°C | T | 1.69 | 0.86 | 2.48 | 5.77 | 4.98 | 1.07 | 37.19 |
| 12 | 26°C | V | 1.45 | 1.03 | 2.79 | 5.45 | 5.03 | 0.81 | 34.86 |
| 12 | 26°C | V | 1.46 | 0.71 | 2.26 | 5.81 | 5.81 | 0.90 | 36.36 |
| 12 | 28°C | T | 2.19 | 1.34 | 2.14 | 3.53 | 6.71 | 1.83 | 31.63 |
| 12 | 28°C | T | 2.14 | 1.08 | 2.20 | 3.57 | 7.53 | 1.70 | 26.96 |
| 12 | 28°C | T | 2.39 | 1.02 | 2.28 | 4.04 | 6.58 | 1.63 | 26.87 |
| 12 | 28°C | V | 2.18 | 0.98 | 2.67 | 3.70 | 7.04 | 1.97 | 28.53 |
| 12 | 28°C | V | 2.10 | 1.28 | 2.05 | 4.11 | 6.50 | 1.76 | 30.11 |
| 25 | 18°C | T | 0.99 | 0.91 | 0.96 | 0.98 | 1.00 | 1.15 | 22.87 |
| 25 | 18°C | T | 0.92 | 0.99 | 0.94 | 0.96 | 1.01 | 0.98 | 24.26 |
| 25 | 18°C | V | 1.15 | 1.17 | 1.05 | 1.08 | 1.03 | 0.94 | 19.58 |
| 25 | 18°C | V | 0.95 | 0.98 | 0.88 | 1.04 | 1.05 | 0.89 | 23.64 |
| 25 | 18°C | V | 0.99 | 0.95 | 1.17 | 0.94 | 0.91 | 1.04 | 22.90 |
| 25 | 24°C | T | 1.17 | 1.04 | 1.28 | 1.78 | 1.40 | 1.12 | 24.10 |
| 25 | 24°C | T | 1.29 | 1.01 | 1.03 | 1.85 | 1.49 | 1.23 | 27.35 |
| 25 | 24°C | V | 1.10 | 1.18 | 0.99 | 1.68 | 1.13 | 1.29 | 23.37 |
| 25 | 24°C | V | 1.13 | 0.98 | 1.08 | 2.06 | 1.27 | 1.11 | 26.67 |
| 25 | 24°C | V | 1.16 | 1.04 | 1.07 | 1.89 | 1.52 | 0.90 | 25.50 |
| 25 | 26°C | T | 1.96 | 2.64 | 2.51 | 2.87 | 2.36 | 1.75 | 30.61 |
| 25 | 26°C | T | 2.45 | 3.04 | 2.28 | 3.68 | 2.61 | 1.66 | 30.98 |
| 25 | 26°C | T | 2.10 | 2.97 | 2.49 | 3.43 | 2.67 | 1.80 | 36.46 |
| 25 | 26°C | V | 2.11 | 2.50 | 2.42 | 3.06 | 2.47 | 1.98 | 32.91 |
| 25 | 26°C | V | 2.03 | 2.90 | 2.95 | 3.46 | 2.73 | 1.61 | 34.04 |
| 25 | 28°C | T | 0.98 | 0.64 | 5.12 | 2.40 | 2.53 | 2.19 | 29.30 |
| 25 | 28°C | T | 1.11 | 0.53 | 4.50 | 2.14 | 2.94 | 1.89 | 28.50 |
| 25 | 28°C | V | 1.40 | 0.57 | 4.48 | 2.23 | 2.62 | 1.68 | 30.09 |
| 25 | 28°C | V | 1.05 | 0.60 | 4.63 | 2.16 | 2.83 | 1.93 | 26.95 |
| 25 | 28°C | V | 1.12 | 0.81 | 4.27 | 1.82 | 2.43 | 1.96 | 30.65 |
