# Supplementary material for: Cell death and antioxidant responses in Mytilus galloprovincialis under heat stress: Evidence of genetic loci potentially associated with thermal resilience
Source: PLoS One. 2025 Apr 23;20(4):e0321682. doi: 10.1371/journal.pone.0321682 (PMC12017574; doi:10.1371/journal.pone.0321682)
Supplement: S5 Table — Statistically significant differences (p < 0.05) are indicated with bold and an asterisk (*). (DOCX) [file pone.0321682.s005.docx]

| Position | Genotypes | Susceptible (%) | Resilient (%) | χ^2^ | *p* | Alleles | Susceptible (%) | Resilient (%) | χ^2^ | *p* |
| --- | --- | --- | --- | --- | --- | --- | --- | --- | --- | --- |
| *fadd* | | | | | | | | | | |
| cds-544 | G/G | 60 | 30 | 3.88 | 0.143 | G  A | 75  25 | 52.5  47.5 | 4.38 | **0.036*** |
|  | G/A | 30 | 45 |  |  |  |  |  |  |  |
|  | A/A | 10 | 25 |  |  |  |  |  |  |  |
| cds-550 | A/A | 42.1 | 47.4 | 0.5 | 0.777 | A  T | 52.6  47.4 | 60.5  39.5 | 0.48 | 0.487 |
|  | A/T | 21.1 | 26.3 |  |  |  |  |  |  |  |
|  | T/T | 36.3 | 26.3 |  |  |  |  |  |  |  |
| *Cu-Zn sod* | | | | | | | | | | |
| cds-195 | T/T | 94.7 | 38.8 | 11.1 | **0.003*** | T  A | 94.7  5.3 | 41.7  58.3 | 24.3 | **8.22 x 10^-7^*** |
|  | A/T | 0 | 5.6 |  |  |  |  |  |  |  |
|  | A/A | 5.3 | 55.6 |  |  |  |  |  |  |  |
| cds-285 | T/T | 63.2 | 44.5 | 7.59 | **0.022*** | T  G | 81.6  18.4 | 55.6  44.4 | 5.84 | **0.015*** |
|  | T/G | 36.8 | 22.2 |  |  |  |  |  |  |  |
|  | G/G | 0 | 33.3 |  |  |  |  |  |  |  |
| *catalase* | | | | | | | | | | |
| cds-1198 | C/C | 64.7 | 76.5 | 3.83 | 0.147 | C  T | 79.4  20.6 | 79.4  20.6 | 0 | 1 |
|  | C/T | 29.4 | 5.9 |  |  |  |  |  |  |  |
|  | T/T | 5.9 | 17.6 |  |  |  |  |  |  |  |

**S5 Table**. **The percentages of the different alleles and genotypes in resilient and susceptible mussels and χ^2^ and *p* values from the chi-squared tests on their distribution among resilient and susceptible individuals.** Statistically significant differences (*p* < 0.05) are indicated with bold and an asterisk (*).
