# Supplementary material for: Cell death and antioxidant responses in Mytilus galloprovincialis under heat stress: Evidence of genetic loci potentially associated with thermal resilience
Source: PLoS One. 2025 Apr 23;20(4):e0321682. doi: 10.1371/journal.pone.0321682 (PMC12017574; doi:10.1371/journal.pone.0321682)
Supplement: S6 Table — For the Brown-Forsythe’s test the test statistic, degrees of freedom (numerator, denominator), and p-value are shown. For ANOVA the test statistic, p-value, and R2 are shown along with the p-values from Tukey’s multiple post-hoc comparisons among treatments. (DOCX) [file pone.0321682.s006.docx]

| **bax**  **S6 Table.** **Results of one-way ANOVA and Tukey's post-hoc comparisons, along with Brown-Forsythe test results for homogeneity of variances.** For the Brown-Forsythe’s test the test statistic, degrees of freedom (numerator, denominator), and *p*-value are shown. For ANOVA the test statistic, *p*-value, and R^2^ are shown along with the *p*-values from Tukey’s multiple post-hoc comparisons among treatments. | | | | | | | | |
| --- | --- | --- | --- | --- | --- | --- | --- | --- |
| **Day 1** | | **Day 3** | | **Day 12** | | **Day 25** | | |
| **Brown-Forsythe test** | | **Brown-Forsythe test** | | **Brown-Forsythe test** | | **Brown-Forsythe test** | | |
| F (DFn, DFd) | p-value | F (DFn, DFd) | p-value | F (DFn, DFd) | p-value | F (DFn, DFd) | p-value | |
| 1.11 (3, 16) | 0.37 | 0.38 (3, 16) | 0.76 | 0.18 (3, 16) | 0.90 | 0.52 (3, 16) | 0.67 | |
| **ANOVA + Tukey’s post hoc** | | **ANOVA + Tukey’s post hoc** | | **ANOVA + Tukey’s post hoc** | | **ANOVA + Tukey’s post hoc** | | |
| F statistic | 12.57 | F statistic | 61.97 | F statistic | 120.1 | F statistic | | 72.66 |
| p-value | 0.0002 | p-value | <0.0001 | p-value | <0.0001 | p-value | | <0.0001 |
| R squared | 0.7021 | R squared | 0.9208 | R squared | 0.9575 | R squared | | 0.9316 |
| **Comparisons** | **Adj. p value** | **Comparisons** | **Adj. p value** | **Comparisons** | **Adj. p value** | **Comparisons** | | **Adj. p value** |
| 18°C vs. 24°C | 0.0031 | 18°C vs. 24°C | <0.0001 | 18°C vs. 24°C | 0.834 | 18°C vs. 24°C | | 0.2393 |
| 18°C vs. 26°C | 0.0019 | 18°C vs. 26°C | <0.0001 | 18°C vs. 26°C | <0.0001 | 18°C vs. 26°C | | <0.0001 |
| 18°C vs. 28°C | 0.0002 | 18°C vs. 28°C | 0.2582 | 18°C vs. 28°C | <0.0001 | 18°C vs. 28°C | | 0.4434 |
| 24°C vs. 26°C | 0.9945 | 24°C vs. 26°C | 0.0003 | 24°C vs. 26°C | 0.0002 | 24°C vs. 26°C | | <0.0001 |
| 24°C vs. 28°C | 0.4555 | 24°C vs. 28°C | 0.0006 | 24°C vs. 28°C | <0.0001 | 24°C vs. 28°C | | 0.9705 |
| 26°C vs. 28°C | 0.5988 | 26°C vs. 28°C | <0.0001 | 26°C vs. 28°C | <0.0001 | 26°C vs. 28°C | | <0.0001 |
| **bcl2** | | | | | | | | |
| **Day 1** | | **Day 3** | | **Day 12** | | **Day 25** | | |
| **Brown-Forsythe test** | | **Brown-Forsythe test** | | **Brown-Forsythe test** | | **Brown-Forsythe test** | | |
| F (DFn, DFd) | p-value | F (DFn, DFd) | p-value | F (DFn, DFd) | p-value | F (DFn, DFd) | | p-value |
| 0.63 (3, 16) | 0.6 | 1.5 (3, 16) | 0.25 | 0.28 (3, 16) | 0.83 | 1.65 (3, 16) | | 0.21 |
| **ANOVA + Tukey’s post hoc** | | **ANOVA + Tukey’s post hoc** | | **ANOVA + Tukey’s post hoc** | | **ANOVA + Tukey’s post hoc** | | |
| F statistic | 3.043 | F statistic | 13.35 | F statistic | 54.64 | F statistic | | 236.7 |
| p-value | 0.0593 | p-value | 0.0001 | p-value | <0.0001 | p-value | | <0.0001 |
| R squared | 0.3633 | R squared | 0.7146 | R squared | 0.9111 | R squared | | 0.978 |
| **Comparisons** | **Adj. p value** | **Comparisons** | **Adj. p value** | **Comparisons** | **Adj. p value** | **Comparisons** | | **Adj. p value** |
| 18°C vs. 24°C | 0.5296 | 18°C vs. 24°C | 0.6927 | 18°C vs. 24°C | <0.0001 | 18°C vs. 24°C | | 0.9432 |
| 18°C vs. 26°C | 0.1546 | 18°C vs. 26°C | 0.0022 | 18°C vs. 26°C | 0.7408 | 18°C vs. 26°C | | <0.0001 |
| 18°C vs. 28°C | 0.0517 | 18°C vs. 28°C | 0.3479 | 18°C vs. 28°C | 0.4137 | 18°C vs. 28°C | | 0.004 |
| 24°C vs. 26°C | 0.8242 | 24°C vs. 26°C | 0.0207 | 24°C vs. 26°C | <0.0001 | 24°C vs. 26°C | | <0.0001 |
| 24°C vs. 28°C | 0.4801 | 24°C vs. 28°C | 0.0541 | 24°C vs. 28°C | <0.0001 | 24°C vs. 28°C | | 0.0013 |
| 26°C vs. 28°C | 0.9296 | 26°C vs. 28°C | <0.0001 | 26°C vs. 28°C | 0.0809 | 26°C vs. 28°C | | <0.0001 |
| **lc3b** | | | | | | | | |
| **Day 1** | | **Day 3** | | **Day 12** | | **Day 25** | | |
| **Brown-Forsythe test** | | **Brown-Forsythe test** | | **Brown-Forsythe test** | | **Brown-Forsythe test** | | |
| F (DFn, DFd) | p-value | F (DFn, DFd) | p-value | F (DFn, DFd) | p-value | F (DFn, DFd) | | p-value |
| 1 (3, 16) | 0.41 | 1.7 (3, 16) | 0.2 | 1.33 (3, 16) | 0.29 | 0.67 (3, 16) | | 0.57 |
| **ANOVA + Tukey’s post hoc** | | **ANOVA + Tukey’s post hoc** | | **ANOVA + Tukey’s post hoc** | | **ANOVA + Tukey’s post hoc** | | |
| F statistic | 96.22 | F statistic | 160.9 | F statistic | 52.44 | F statistic | | 298.6 |
| p-value | <0.0001 | p-value | <0.0001 | p-value | <0.0001 | p-value | | <0.0001 |
| R squared | 0.9475 | R squared | 0.9679 | R squared | 0.9077 | R squared | | 0.9825 |
| **Comparisons** | **Adj. p value** | **Comparisons** | **Adj. p value** | **Comparisons** | **Adj. p value** | **Comparisons** | | **Adj. p value** |
| 18°C vs. 24°C | 0.7832 | 18°C vs. 24°C | 0.5231 | 18°C vs. 24°C | 0.0002 | 18°C vs. 24°C | | 0.9128 |
| 18°C vs. 26°C | 0.7024 | 18°C vs. 26°C | <0.0001 | 18°C vs. 26°C | <0.0001 | 18°C vs. 26°C | | <0.0001 |
| 18°C vs. 28°C | <0.0001 | 18°C vs. 28°C | <0.0001 | 18°C vs. 28°C | <0.0001 | 18°C vs. 28°C | | <0.0001 |
| 24°C vs. 26°C | 0.2189 | 24°C vs. 26°C | <0.0001 | 24°C vs. 26°C | <0.0001 | 24°C vs. 26°C | | <0.0001 |
| 24°C vs. 28°C | <0.0001 | 24°C vs. 28°C | <0.0001 | 24°C vs. 28°C | 0.0187 | 24°C vs. 28°C | | <0.0001 |
| 26°C vs. 28°C | <0.0001 | 26°C vs. 28°C | <0.0001 | 26°C vs. 28°C | 0.0562 | 26°C vs. 28°C | | <0.0001 |
| **fadd** | | | | | | | | |
| **Day 1** | | **Day 3** | | **Day 12** | | **Day 25** | | |
| **Brown-Forsythe test** | | **Brown-Forsythe test** | | **Brown-Forsythe test** | | **Brown-Forsythe test** | | |
| F (DFn, DFd) | p-value | F (DFn, DFd) | p-value | F (DFn, DFd) | p-value | F (DFn, DFd) | | p-value |
| 0.86 (3, 16) | 0.48 | 0.54 (3, 16) | 0.65 | 0.7 (3, 16) | 0.56 | 1.61 (3, 16) | | 0.22 |
| **ANOVA + Tukey’s post hoc** | | **ANOVA + Tukey’s post hoc** | | **ANOVA + Tukey’s post hoc** | | **ANOVA + Tukey’s post hoc** | | |
| F statistic | 216 | F statistic | 157.6 | F statistic | 295.4 | F statistic | | 103.2 |
| p-value | <0.0001 | p-value | <0.0001 | p-value | <0.0001 | p-value | | <0.0001 |
| R squared | 0.9759 | R squared | 0.9673 | R squared | 0.9823 | R squared | | 0.9509 |
| **Comparisons** | **Adj. p value** | **Comparisons** | **Adj. p value** | **Comparisons** | **Adj. p value** | **Comparisons** | | **Adj. p value** |
| 18°C vs. 24°C | 0.5246 | 18°C vs. 24°C | 0.6379 | 18°C vs. 24°C | 0.0004 | 18°C vs. 24°C | | <0.0001 |
| 18°C vs. 26°C | <0.0001 | 18°C vs. 26°C | <0.0001 | 18°C vs. 26°C | <0.0001 | 18°C vs. 26°C | | <0.0001 |
| 18°C vs. 28°C | 0.6741 | 18°C vs. 28°C | 0.9997 | 18°C vs. 28°C | <0.0001 | 18°C vs. 28°C | | <0.0001 |
| 24°C vs. 26°C | <0.0001 | 24°C vs. 26°C | <0.0001 | 24°C vs. 26°C | <0.0001 | 24°C vs. 26°C | | <0.0001 |
| 24°C vs. 28°C | 0.9941 | 24°C vs. 28°C | 0.6914 | 24°C vs. 28°C | <0.0001 | 24°C vs. 28°C | | 0.1516 |
| 26°C vs. 28°C | <0.0001 | 26°C vs. 28°C | <0.0001 | 26°C vs. 28°C | <0.0001 | 26°C vs. 28°C | | <0.0001 |
| **Cu-Zn sod** | | | | | | | | |
| **Day 1** | | **Day 3** | | **Day 12** | | **Day 25** | | |
| **Brown-Forsythe test** | | **Brown-Forsythe test** | | **Brown-Forsythe test** | | **Brown-Forsythe test** | | |
| F (DFn, DFd) | p-value | F (DFn, DFd) | p-value | F (DFn, DFd) | p-value | F (DFn, DFd) | | p-value |
| 1.26 (3, 16) | 0.31 | 1.64 (3, 16) | 0.21 | 1.5 (3, 16) | 0.25 | 1.65 (3, 16) | | 0.21 |
| **ANOVA + Tukey’s post hoc** | | **ANOVA + Tukey’s post hoc** | | **ANOVA + Tukey’s post hoc** | | **ANOVA + Tukey’s post hoc** | | |
| F statistic | 368.4 | F statistic | 152.8 | F statistic | 334 | F statistic | | 147.7 |
| p-value | <0.0001 | p-value | <0.0001 | p-value | <0.0001 | p-value | | <0.0001 |
| R squared | 0.9857 | R squared | 0.9663 | R squared | 0.9843 | R squared | | 0.9651 |
| **Comparisons** | **Adj. p value** | **Comparisons** | **Adj. p value** | **Comparisons** | **Adj. p value** | **Comparisons** | | **Adj. p value** |
| 18°C vs. 24°C | 0.3822 | 18°C vs. 24°C | <0.0001 | 18°C vs. 24°C | 0.674 | 18°C vs. 24°C | | 0.0097 |
| 18°C vs. 26°C | <0.0001 | 18°C vs. 26°C | <0.0001 | 18°C vs. 26°C | <0.0001 | 18°C vs. 26°C | | <0.0001 |
| 18°C vs. 28°C | <0.0001 | 18°C vs. 28°C | <0.0001 | 18°C vs. 28°C | <0.0001 | 18°C vs. 28°C | | <0.0001 |
| 24°C vs. 26°C | <0.0001 | 24°C vs. 26°C | 0.0909 | 24°C vs. 26°C | <0.0001 | 24°C vs. 26°C | | <0.0001 |
| 24°C vs. 28°C | <0.0001 | 24°C vs. 28°C | <0.0001 | 24°C vs. 28°C | <0.0001 | 24°C vs. 28°C | | <0.0001 |
| 26°C vs. 28°C | <0.0001 | 26°C vs. 28°C | <0.0001 | 26°C vs. 28°C | <0.0001 | 26°C vs. 28°C | | 0.7302 |
| **sqrt (catalase)** | | | | | | | | |
| **Day 1** | | **Day 3** | | **Day 12** | | **Day 25** | | |
| **Brown-Forsythe test** | | **Brown-Forsythe test** | | **Brown-Forsythe test** | | **Brown-Forsythe test** | | |
| F (DFn, DFd) | p-value | F (DFn, DFd) | p-value | F (DFn, DFd) | p-value | F (DFn, DFd) | | p-value |
| 0.25 (3, 16) | 0.86 | 0.37 (3, 16) | 0.77 | 0.13 (3, 16) | 0.93 | 0.18 (3, 16) | | 0.9 |
| **ANOVA + Tukey’s post hoc** | | **ANOVA + Tukey’s post hoc** | | **ANOVA + Tukey’s post hoc** | | **ANOVA + Tukey’s post hoc** | | |
| F statistic | 50.97 | F statistic | 74.65 | F statistic | 54.63 | F statistic | | 48.88 |
| p-value | <0.0001 | p-value | <0.0001 | p-value | <0.0001 | p-value | | <0.0001 |
| R squared | 0.9053 | R squared | 0.9333 | R squared | 0.9111 | R squared | | 0.9016 |
| **Comparisons** | **Adj. p value** | **Comparisons** | **Adj. p value** | **Comparisons** | **Adj. p value** | **Comparisons** | | **Adj. p value** |
| 18°C vs. 24°C | 0.7789 | 18°C vs. 24°C | <0.0001 | 18°C vs. 24°C | 0.8117 | 18°C vs. 24°C | | 0.5166 |
| 18°C vs. 26°C | 0.8276 | 18°C vs. 26°C | <0.0001 | 18°C vs. 26°C | 0.5582 | 18°C vs. 26°C | | <0.0001 |
| 18°C vs. 28°C | <0.0001 | 18°C vs. 28°C | 0.0001 | 18°C vs. 28°C | <0.0001 | 18°C vs. 28°C | | <0.0001 |
| 24°C vs. 26°C | 0.3071 | 24°C vs. 26°C | <0.0001 | 24°C vs. 26°C | 0.1608 | 24°C vs. 26°C | | <0.0001 |
| 24°C vs. 28°C | <0.0001 | 24°C vs. 28°C | <0.0001 | 24°C vs. 28°C | <0.0001 | 24°C vs. 28°C | | <0.0001 |
| 26°C vs. 28°C | <0.0001 | 26°C vs. 28°C | 0.9881 | 26°C vs. 28°C | <0.0001 | 26°C vs. 28°C | | 0.2951 |
| **TBARS (fold change)** | | | | | | | | |
| **Day 1** | | **Day 3** | | **Day 12** | | **Day 25** | | |
| **Brown-Forsythe test** | | **Brown-Forsythe test** | | **Brown-Forsythe test** | | **Brown-Forsythe test** | | |
| F (DFn, DFd) | p-value | F (DFn, DFd) | p-value | F (DFn, DFd) | p-value | F (DFn, DFd) | | p-value |
| 0.15 (3, 16) | **0.92** | 0.17 (3, 16) | 0.91 | 0.13 (3, 16) | 0.93 | 0.42 (3, 16) | | 0.73 |
| **ANOVA + Tukey’s post hoc** | | **ANOVA + Tukey’s post hoc** | | **ANOVA + Tukey’s post hoc** | | **ANOVA + Tukey’s post hoc** | | |
| F statistic | 18.62 | F statistic | 1.545 | F statistic | 23.26 | F statistic | | 28.75 |
| p-value | <0.0001 | p-value | 0.2415 | p-value | <0.0001 | p-value | | <0.0001 |
| R squared | 0.7774 | R squared | 0.2246 | R squared | 0.8135 | R squared | | 0.8435 |
| **Comparisons** | **Adj. p value** | **Comparisons** | **Adj. p value** | **Comparisons** | **Adj. p value** | **Comparisons** | | **Adj. p value** |
| 18°C vs. 24°C | 0.0608 | 18°C vs. 24°C | 0.8198 | 18°C vs. 24°C | <0.0001 | 18°C vs. 24°C | | 0.1248 |
| 18°C vs. 26°C | 0.9576 | 18°C vs. 26°C | 0.7326 | 18°C vs. 26°C | 0.0001 | 18°C vs. 26°C | | <0.0001 |
| 18°C vs. 28°C | <0.0001 | 18°C vs. 28°C | 0.8198 | 18°C vs. 28°C | 0.9978 | 18°C vs. 28°C | | 0.0003 |
| 24°C vs. 26°C | 0.1504 | 24°C vs. 26°C | 0.9984 | 24°C vs. 26°C | 0.9886 | 24°C vs. 26°C | | <0.0001 |
| 24°C vs. 28°C | 0.0058 | 24°C vs. 28°C | 0.336 | 24°C vs. 28°C | 0.0001 | 24°C vs. 28°C | | 0.0321 |
| 26°C vs. 28°C | <0.0001 | 26°C vs. 28°C | 0.2651 | 26°C vs. 28°C | 0.0003 | 26°C vs. 28°C | | 0.022 |
